# Markers for the non-invasive diagnosis of mesothelioma: a systematic review

**DOI:** 10.1038/bjc.2011.104

**Published:** 2011-03-29

**Authors:** S van der Bij, E Schaake, H Koffijberg, J A Burgers, B A J M de Mol, K G M Moons

**Affiliations:** 1 Julius Center for Health Sciences and Primary Care, University Medical Center Utrecht, Stratenum 6.131, PO Box 85500, 3508 GA Utrecht, The Netherlands; 2 Department of Thoracic Oncology, The Netherlands Cancer Institute - Antoni van Leeuwenhoek Hospital, Plesmanlaan 121 1066 CX, Amsterdam, The Netherlands; 3 Department of Cardiothoracic Surgery, Academic Medical Centre, Meibergdreef 9, Amsterdam 1105 AZ, The Netherlands; 4 Department of Biomedical Engineering, Eindhoven University of Technology, Den Dolech 2, Eindhoven 5600 MB, The Netherlands

**Keywords:** mesothelioma, diagnosis, biomarkers, cytology

## Abstract

**Background:**

Numerous markers have been evaluated to facilitate the non-invasive diagnostic work-up of mesothelioma. The purpose of this study was to conduct a structured review of the diagnostic performance of non-invasive marker tests for the detection of mesothelioma in patients with suspected mesothelioma.

**Methods:**

Studies on the diagnostic accuracy of serum and cytological markers published till 31 December 2009, available in either PUBMED or Embase, to detect or exclude the presence of mesothelioma were extracted. Study quality was assessed with use of the Quadas criteria.

**Results:**

In total, 82 articles were included in this systemic review. Overall, quality of the incorporated studies to address our objective was poor. The most frequently studied immunohistochemical markers for cytological analysis were EMA, Ber-Ep4, CEA, and calretinin. The most frequently investigated serum marker was soluble mesothelin-related protein (SMRP). The markers CEA, Ber-EP4, and calretinin were most valuable in discriminating mesothelioma from other malignant diseases. Markers EMA and SMRP were most valuable in discriminating mesothelioma from non-malignant diseases. No marker performed well in discriminating between mesothelioma and all other diseases.

**Conclusion:**

Currently, there is only limited evidence to properly assess the value of non-invasive marker tests in the diagnosis of mesothelioma. Studies were of limited value to address our objective and results showed considerable unexplained study heterogeneity.

The diagnosis of mesothelioma is not straightforward. The symptoms are nonspecific, and only in experienced centres, pleural fluid cytology is a reliable diagnostic tool. Hence, most patients ultimately require invasive procedures such as core-needle or open biopsy, or video-assisted thoracoscopy to facilitate histological examination as ‘gold’ standard for diagnosis (Renshaw *et al*, 1997; Fletcher and Clark, 2007; Fassina *et al*, 2008). However, a biopsy may complicate subsequent disease management by seeding tumor cells or may be unfeasible because of poor condition of the patient. Therefore, it would be valuable to have non-invasive diagnostic procedures that accurately confirm or exclude the diagnosis of mesothelioma.

Accordingly, innumerable non-invasive markers have emerged, based on the increasing understanding of the molecular and biological pathways of mesothelioma, and studied in numerous studies. These include many immunohistochemical markers that have been tested for their property to establish the diagnosis of mesothelioma on cytological grounds (Lyons-Boudreaux *et al*, 2008). Promising other tests are genetic markers and serum markers such as soluble mesothelin-related protein (SMRP) and megakaryocyte potentiating factor (Pass *et al*, 2005; Holloway *et al*, 2006; Scherpereel *et al*, 2006; Creaney *et al*, 2008).

However, estimated diagnostic accuracy of identical markers varies widely between studies. Therefore, it remains unclear which marker has a superior performance. Nevertheless, several markers have already entered the market and are used in clinical practice. In contrast, others disappeared after initial promising results. As a result, current diagnostic strategies for mesothelioma-involving markers are likely to be suboptimal. Therefore, we conducted a systematic review to summarise the literature on the diagnostic accuracy of serum and cytological markers for the diagnosis of mesothelioma.

## Materials and methods

### Search strategy

The systematic search addressed articles with information on markers in serum and effusions to include or exclude the presence of mesothelioma published till 31 December 2009. The search was carried out with Medline and PUBMED (Supplementary Appendix 1 for search strategy). Duplicates from Medline and Embase were deleted automatically and manually with Reference Manager v11 (Thomson Reuters, New York, NY, USA).

### Markers (index tests)

To facilitate the analysis, and to allow a more appropriate comparison between the studies, we divided the non-invasive markers into four groups: serum markers; effusion markers, that is, pleural and peritoneal fluid markers; immunohistochemical markers used for cytological analysis of effusion samples; and genetic markers.

### Selection

To be eligible for inclusion, studies had to fulfil all of the following criteria:

1. The study should be an original report in English (i.e., letters, editorials, case reports, tutorials, reviews, and non-English studies were excluded).
2. The study should assess the ability of one or more markers to detect or exclude the presence of mesothelioma, and only involving non-invasive marker tests. Studies in which marker tests were assessed in tissue biopsy samples, pelvic washings, or more than 10% fine-needle aspirates were not included.
3. The diagnosis of mesothelioma had to be confirmed on at least cytology and/or histology.
4. The study should have a minimal sample size of 10 mesothelioma patients.
5. The study should provide sufficient data to (re)construct a two-by-two contingency table to estimate the marker's diagnostic accuracy.

Studies reporting ⩾10% more specimens than study patients, indicating that more than one specimen per patient was used, were excluded. Furthermore, studies investigating markers in high-risk study populations for screening or surveillance purposes were excluded.

The article selection was performed in two consecutive phases: title and abstract assessment (one reviewer, SB) and full-article assessment (two independent reviewers, SB and ES).

### Data extraction

If a study was included, the two reviewers independently extracted the following elements from the article: overall study characteristics, for example, author(s), institution, date of publication, recruitment setting, study design and study years; participant characteristics, for example, description of the mesothelioma patients and comparison group; details of the index marker test including the positive *vs* negative cut-off value; and type of reference test used to confirm the presence or absence of mesothelioma.

The number of true positives (TP), false positives (FP), true negatives (TN) and false negatives (FN) were extracted and used to construct a two-by-two table, if possible, separately for each comparison group. Comparison groups were summarised to either other malignancies or no malignancies, which could include also healthy participants. If more than one cut-off value was used, we selected the value closest to the cut-off corresponding with 95% specificity (avoiding FPs as much as possible). For immunohistochemical markers, we selected the value closest to the 10% cut-off according to the percentage of cells exhibiting staining (as it is a frequently used value in immunocytology and implies that samples were considered positive for the marker if at least 10% of malignant mesothelioma cells were immunohistochemically stained). Data of the diagnostic value of a combination of markers were not extracted.

Discrepancies between the two reviewers were resolved by consensus. If needed, a third and a fourth reviewer (HK, KGMM) resolved the remaining discrepancies. When studies with overlapping data sets were published, preference was given to those studies, which had the highest number of mesothelioma patients or used malignancy as a comparison group (which better reflects clinical practice). If a study evaluated various markers and results of a subset of these markers were published in a more recent study, then only the results of the duplicate markers were excluded from the first study.

### Quality assessment

The methodological quality of each included study was independently assessed by the two reviewers using the Quadas instrument (see Supplementary Appendix 2), a widely accepted and validated tool for the quality assessment of diagnostic accuracy studies in systematic reviews (Whiting *et al*, 2003). In case of doubt, a third or fourth reviewer was consulted (HK and KGMM).

### Data synthesis

Results were summarised as per type of marker and as per comparison group (i.e., other malignancies or no malignancies). Markers reported in at least six studies were described more comprehensively. As is common in diagnostic systematic reviews and meta-analysis, we used sensitivity and specificity as our primary measures of association. Sensitivity was calculated by dividing TP by (TP+FN) and specificity by dividing TN by (FP+TN) from the (re)constructed two-by-two tables. Associated 95% confidence intervals (CIs) were assessed using the Wilson score method (Newcombe, 1998). To graphically present the results, estimates of sensitivity and specificity of a single marker across studies were summarised in a receiver-operating characteristic (ROC) graph, plotting the markers’ sensitivity on the *y* axis against the 1−specificity on the *x* axis. When different studies on the same biomarker shows different sensitivity and specificity, it does not necessarily mean that the results are different or heterogeneous; they might simply have used a different (explicit or implicit) cut-off value for marker positivity. As with a change in cut-off value, the sensitivity and specificity commonly increase or decrease in opposite directions (negative correlation), the ROC curve for such marker should show a concave, shoulder-like pattern. For each marker with different sensitivities and specificities plotted in ROC space, we quantify whether this could be explained by such threshold effect by estimating the (negative) correlation between sensitivity and specificity. This was performed on the logit scale using the bivariate model (Reitsma *et al*, 2005). All analyses were performed in SAS statistical packages, version 9.1 (SAS Institute Inc., Cary, NC, USA).

## Results

### Search results

**Figure 1:**
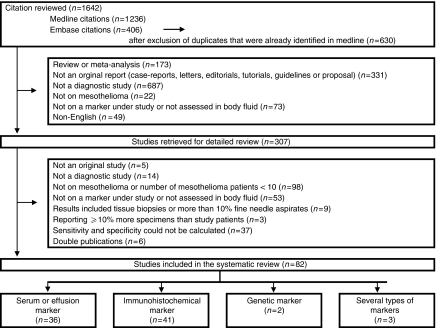
Flowchart of the selection of the relevant articles. Serum and effusion markers include tests to detect serum and effusion marker levels; immunohistochemical markers include marker tests used for cytological analysis of effusion samples; genetic markers include polymerase chain reaction tests to detect specific gene expressions or fluorescence *in situ* hybridisation (FISH) tests to detect gene deletions with the use of specialised gene probes.

Our search provided 1642 hits, of which 307 were eligible for inclusion, based on title and abstract. After assessment of the full-text articles, 224 articles were discarded for various reasons (Figure 1). Thus, this review included 82 articles (see Supplementary Appendix 3): 36 articles that evaluated serum or effusion markers, 41 on immunohistochemical markers, 2 studies on genetic markers, and 3 studies on different types of markers. Most immunohistochemical studies included epitheloid and biphasic mesotheliomas.

### Study quality

**Figure 2:**
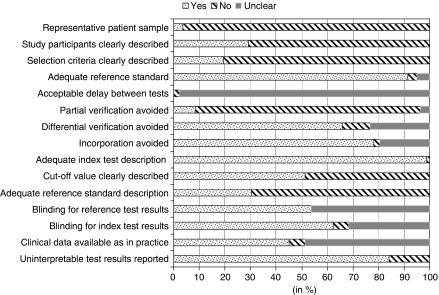
Summary of quality of the included studies according to the Quadas criteria (see Supplementary Appendix).

The methodological quality of the studies with focus on the objective of this review was generally poor and is shown in Figure 2, with specific details in Table 1 (references to these studies are prefaced by an ‘r’ and listed in Supplementary Appendix 3). Only three articles were identified that adequately selected a representative cohort of consecutive patients suspected for mesothelioma.^r3,r22,r76^ Of these, two articles were on the basis of one prospective French study.^r3,r22^ Other studies used a case–control design (*n*=70), or a cohort of patients with pleural effusions (*n*=9). Owing to these designs, nearly all studies (88%) suffered from the well-described and problematic disease-verification bias (Begg and Greenes, 1983; Lijmer *et al*, 1999; Mol *et al*, 2003; Whiting *et al*, 2004; Rutjes *et al*, 2005; Biesheuvel *et al*, 2008). Furthermore, most studies did not have an adequate description of the patient-selection procedure, characteristics of the study participants, the reference standard, and the used cut-off value of the marker. The time between index test (marker) and reference test as well as the availability of other clinical data (as is commonly encountered in practice) were also poorly reported. Blinding for the results of the marker (index test) when interpreting the reference test (and vice versa) was fulfilled in about 55% of the studies.

### Investigated markers

Supplementary Appendix 4 provides a complete summary of the performance of all markers, across the included studies. In total, 54 immunohistochemical markers, 21 serum markers, 12 effusion markers, and 1 genetic marker were identified. The most frequently evaluated immunohistochemical marker was EMA followed by BER-EP4, CEA, and calretinin (Supplementary Appendix 4, Table 2.3). Among serum markers, the most frequently investigated were SMRP and CEA, (Supplementary Appendix, Table 2.1) and among effusion markers CEA, CA15-3, HA, and SMRP (Supplementary Appendix 4, Table 2.2). Results on genetic markers were sparse (Supplementary Appendix 4, Table 2.4). The number of eligible papers allowed a closer evaluation of SMRP in serum and CEA in effusion, as well as the immunohistochemical value of CEA, Ber-EP4, calretinin, and EMA.

**Figure 3:**
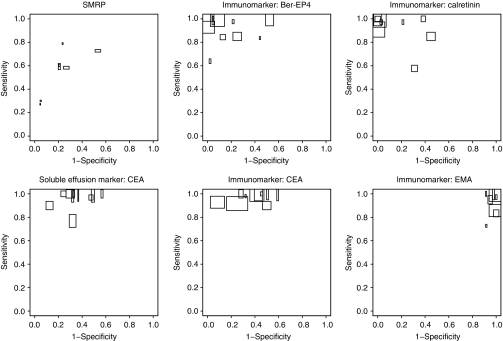
Sensitivity against 1-specificity in ROC space to discriminate mesothelioma from other malignant diseases. The height of the blocks is proportional to the reciprocal of the number of mesothelioma patients (mesothelioma yes subjects) and the width of the blocks is proportional to the reciprocal of the number of patients with other malignant diseases (mesothelioma no subjects).

**Figure 4:**
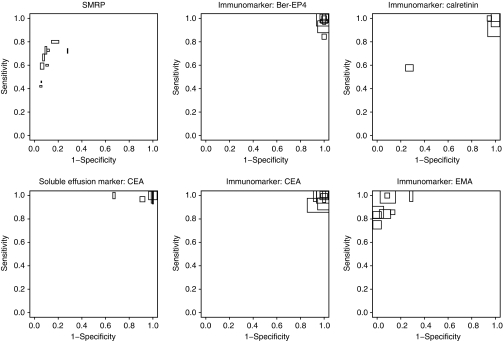
Sensitivity against 1-specificity in ROC space to discriminate mesothelioma from non-malignancy. The height of the blocks is proportional to the reciprocal of the number of mesothelioma patients (mesothelioma yes subjects) and the width of the blocks is proportional to the reciprocal of the number of non-malignant patients (mesothelioma no subjects).

Figures 3 and 4 show the ROC space plots for the SMRP in serum and CEA in effusions, and the immunohistochemical markers Ber-Ep4, CEA, EMA, and calretinin. In Figure 3, their performance to discriminate mesothelioma from other malignant diseases is shown, and in Figure 4, the performance to discriminate mesothelioma from non-malignancies. From these figures a clear threshold effect seems apparent for SMRP, meaning that the variation between studies is probably due to differences in the applied positivity threshold. Studies with a higher threshold mostly produced higher sensitivities and lower specificities. This finding is supported by the significant negative correlations between the logit sensitivity and logit specificity (−0.95, 95% CI: −0.99 to −0.27 in Figure 3 and −1.00, 95% CI: −1.00 to −0.99 in Figure 4). Overall, SMRP levels were lower among sarcomatoid mesothelioma compared with the other types (data not shown).

In all CEA studies, effusion CEA levels lower than 40 ng ml^−1^ were compatible with both non-malignancy and mesothelioma. Discrimination between mesothelioma and non-malignancy, based on CEA levels, was therefore poor (Figure 4). The levels of CEA among other malignancies were in general higher than in mesothelioma patients. Figure 3 shows that the specificity of CEA (i.e., the proportion of patients with other malignant diseases above a specific cut-off point) varied widely among studies and ranged from 43 (95% CI: 33–54) to 88% (95% CI: 69–96). These differences could only partly be explained by differences in the applied cut-off value (correlation was not significant), and by the type of other malignancies included in the control group.

The immunohistochemical markers Ber-EP4, CEA, and calretinin can be useful in discriminating mesothelioma from other malignant diseases (Figure 3), whereas EMA can be useful in discriminating mesothelioma from non-malignant diseases (Figure 4). Specificity of Ber-Ep4 and CEA was more heterogeneous than sensitivity and sensitivity was, in general, high (Figure 3). For calretinin, the sensitivity ranged from 85 to 100%, except for the study of Simsir *et al*^r61^. In that study^r65^ calretinin staining was much lower among mesothelioma and benign samples.

The immunohistochemical marker EMA had a positive cytoplasmic or membranous staining in the majority of the papers, ranging from 73 to 100% among mesothelioma patients and from 91 to 100% among other malignant diseases. Four studies^r39,r42,r75,r82^ made a distinction in staining pattern as well, showing that a membranous staining EMA pattern was mainly observed in mesothelioma patients (55–92%) and not in other malignant diseases (<20%) (Supplementary Appendix 4, Table 2.3). Discriminating mesothelioma from non-malignant diseases based on EMA provided high sensitivity and specificity (Figure 4). For EMA the correlation between logit sensitivity and specificity was nonsignificant in Figure 4 (−0.56 (95% CI: −0.92 to –0.30)).

### Direct marker comparisons

Some of the studies evaluated multiple markers on the same patients. Two studies^r42,r48^ evaluated both the accuracy of calretinin and CEA. To discriminate mesothelioma from other malignant diseases, both the studies showed that specificity was higher for calretinin (in both studies: 100%) compared with CEA (in both about 58%). Corresponding sensitivities were 91 and 100% for calretinin and 100% (in both studies) for CEA.

Three studies^r39,r48,r80^ in which calretinin and Ber-Ep4 were assessed showed that calretinin was a better discriminator than Ber-EP4, whereas one other study ^r42^ showed a similar performance of both markers.

Seven other studies directly compared the immunohistochemical markers CEA and Ber-EP4.^r46,r48,r64,r67,r73,r82^ Sensitivity values were highest for CEA, and in five of the seven studies^r42,r46,r64,r67,r73^ Ber-Ep4 provided the highest specificity.

No robust conclusion could be drawn on the relative performance of markers across comparative studies because of large differences in study methods and heterogeneity of the results (Table 1, Supplementary Appendix 4, Table 2.3).

## Discussion

We systematically reviewed all available evidence on the diagnostic performance of markers in serum, pleural fluid, and ascites, used to non-invasively discriminate mesothelioma from non-mesothelioma disorders. Numerous markers have been assessed. SMRP, CEA, Ber-EP4, calretinin, and EMA were studied most frequently. We found that the majority of studies had an exploratory design and as such showed a rather poor reporting and low quality as scored by the Quadas instrument for assessing methodological quality of individual studies in diagnostic reviews. Nevertheless, despite this, our analyses indicate that the most valuable markers appear to be CEA, Ber-EP4, and calretinin to discriminate mesothelioma from other malignant diseases. The markers EMA and SMRP were most valuable in discriminating mesothelioma from non-malignant diseases. None of the markers performed well to differentiate mesothelioma from all other diseases.

Furthermore, all the immunohistochemical markers, especially CEA, are of value in exclusion of mesothelioma as sensitivity was in general high. So, positive staining for CEA and Ber-EP4 and negative staining for EMA and calretinin are reassuring that a patient does not have mesothelioma. The specificity of these markers varied and depended on the comparison group and therefore the differential diagnosis. The marker SMRP might be of value confirming the diagnosis mesothelioma when a high cut-off-value is applied (resulting in high specificity).

Our data involved the markers used for cytological examination of pleural fluid and ascites, as well as markers used to test serum, and pleural fluid and ascites levels. To our knowledge, no comprehensive systematic literature search on immunohistochemical markers in the cytological diagnosis of mesothelioma has been performed previous to this study. Recently, a meta-analysis was published on the diagnostic performance of serum SMRP only (Luo *et al*, 2010). Notwithstanding large differences in the methods of data extraction, the inferences of that review were consistent with ours. Still, we come to another conclusion about the study quality. Other meta-analyses on effusion markers focused on differentiating benign from malignant diseases in general, and as such are not directly comparable with our review as our focus was to quantify the diagnostic accuracy of these markers for discriminating mesothelioma from non-mesothelioma (Liang *et al*, 2008; Shi *et al*, 2008). Other reviews in this field did not at all perform a systematic search, and might, thus, be liable to selection bias in terms of included studies (Scherpereel and Lee, 2007; Greillier *et al*, 2008; Creaney and Robinson, 2009).

To appreciate this systematic review, various issues should be addressed. First, the rather low quality of the eligible studies limits the conclusions about the value of markers in the diagnosis of mesothelioma. Therefore, conform to prevailing guidelines of diagnostic meta-analyses, we explicitly refrained to meta-analyse or pool the sensitivities and specificities of the individual markers. The low quality might be partly explained by including all studies with information on markers for mesothelioma, regardless of their main objective. The design of most studies was exploratory, rather than confirmatory, which is illustrated by the fact that 88 markers were studies in the 82 selected papers. Exclusion of all studies with low-quality scores on the Quadas instrument would have interfered with our main objective to obtain a complete overview of markers, and was therefore not performed. Furthermore, just a few studies had an acceptable quality, and only two studies had a prospective selection of consecutive patients suspected of mesothelioma. Several other studies used a prospective, consecutive patient inclusion, but selected patients on grounds of the presence of pleural effusion, rather than on the initial suspicion of mesothelioma.^r2,r31,r34^ Once pleural effusion is confirmed by imaging, only those patients that are still suspected of mesothelioma after imaging are warranted for further testing for mesothelioma. The most frequently applied design was the case–control design, in a retrospective manner. This design has been criticised for leading to biased estimates of accuracy (Lijmer *et al*, 1999; Mol *et al*, 2003; Whiting *et al*, 2003, 2004; Rutjes *et al*, 2005; Biesheuvel *et al*, 2008). Owing to this high number of case–control studies, we could not validly combine ‘benign and other malignant diseases’ into one control group. Otherwise, overall sensitivity and specificity would have been strongly depended on the distribution of other malignant and non-malignant diseases, included in these studies.

Second, reporting of study details was also poor. For example, some studies explicitly stated that they excluded paucicellular cytological samples, whereas the majority of studies provided no details about which types of other malignant or non-malignant cases were included in the control subjects. Owing to the low quality and poor reporting of study details, we could also not explore study heterogeneity.

Third, we did not assess the diagnostic value of combined markers but focused on the value of single markers instead. Pathological examination of effusion includes the use of several immunohistochemical markers. However, as studies used different combinations of markers we did not have sufficient studies to properly meta-analyse their diagnostic accuracy. Nevertheless, knowledge of the value of individual markers will certainly add to the performance of combined marker sets.

Fourth, we did not search for non-published studies because of the large number of studies identified. Hence, our results may suffer from publication bias. Also, studies, which did not report proportions of patients above or below a certain cut-off value could not be included in our analysis as no two-by-two table could be constructed. This mainly involved studies, which showed no difference in mean or median marker levels among groups.

Finally, head-to-head comparisons are preferred to meta-analytically compare the diagnostic accuracy of markers. Although sufficient studies were performed to evaluate both CEA and Ber-Ep4, no robust conclusion could be drawn on their relative performance because of the heterogeneity of the studies.

Having raised these concerns, the question remains which markers are most suitable for use in clinical practice. The aim of developing serum and cytological markers is to establish a non-invasive diagnosis of mesothelioma to prevent the already weakened patient undergoing invasive tests. In addition, the diagnosis of mesothelioma should be firm to enable a financial compensation, requiring markers to have high specificity. A major advantage of SMRP is that it can be applied by the patient's physician, whereas the use of cytological immunohistochemical markers is reserved for a pathologist. Unfortunately, the diagnostic performance of SMRP alone seems not (yet) high enough for that purpose. The specificity of cytological markers (CEA, Ber-EP4, calretinin) appears to be rather heterogeneous, potentially, because of differences in study quality, marker handling, type of antibody, type of effusion, and patient and sample selection among studies. The immunohistochemical marker EMA will only provide a high specificity when the differential diagnosis is between mesothelioma and reactive mesothelial proliferation. However, the EMA marker was not always 100% specific across the studies. Moreover, the value of markers, in particular immunohistochemical markers, depends on the type of mesothelioma. Sarcomatoid mesothelioma, which accounts for about 15% of all mesotheliomas, shed almost no malignant cells into the fluid-making markers less useful (Husain *et al*, 2009). Most immunohistochemical studies that we scrutinised included only epitheloid and biphasic mesotheliomas. Furthermore, morphology has a major role in the decision-making process when evaluating cytological samples. Unfortunately, the majority of the studies did not consider the (added) value of immunohistochemical staining in relation to morphology.

To date, the vast majority of the studies on mesothelioma markers seem to involve rather early-phase diagnostic studies (using retrospective, case–control type of designs) (Fryback and Thornbury, 1991; Lijmer *et al*, 2009). It seems that the next step in studying the most promising markers, is the conduction of prospective accuracy studies in the proper target population, that is, patients selected on their suspicion of having mesothelioma, rather than on its true presence or absence (Rutjes *et al*, 2005; Biesheuvel *et al*, 2008). Subsequently, the incremental marker value of these markers beyond existing diagnostics such as patient characteristics and previous clinical tests, should be investigated (Riley *et al*, 2009; Moons, 2010). Indeed, these prospective studies are extremely hard to perform by single institutions if the disease under study has incidences as low as that of mesothelioma. Hence, we encourage researchers and physicians to join forces to enhance the proper quantification of the diagnostic accuracy of the most promising markers for mesothelioma. Alternatively, retrospective nested case–control studies could be conducted, which are especially efficient for rare diseases and if human material is stored (Rutjes *et al*, 2005; Biesheuvel *et al*, 2008; Moons, 2010). In these studies both cases and controls can be sampled from a single-source population, typically defined by the initial presentation or suspicion of the patient. This systematic review indicated that promising markers that certainly allow for further validation are SMRP, CEA, EMA, calretinin, and Ber-Ep4. In addition, other markers might be promising, which have not yet been validated in a number of studies, for example TTF-1. Finally, we encourage the improvement of reporting of diagnostic accuracy studies, following the STARD guidelines (Bossuyt *et al*, 2003a, 2003b). Only accurate quantification and reporting of the (added) value of mesothelioma markers will lead to the clinical use of the appropriate markers.

## Figures and Tables

**Table 1:** Study characteristics and quality of included studies (ordered by year of study)

			**Quality assessment^a^**														
**First author-year**	**Study design**	**Index test**	**1a**	**1b**	**2**	**3**	**4**	**5**	**6**	**7**	**8a**	**8b**	**9**	**10**	**11**	**12**	**13**
*Studying serum or effusion markers*																	
Aleman – 2009^r1^	Case–control	SMRP^(e)^	2	2	1	1	3	2	1	1	1	1	2	1	1	1	1
Davies – 2009^r2^	Prospective cohort of consecutive patients with pleural effusion, suspected of pleural malignancy	SMRP^(e)^	2	1	1	1	3	2	1	1	1	1	2	1	1	1	1
Grigoriu – 2009^r3 b^	Cohort of patients with suspected mesothelioma	HA^(es)^	1	1	1	1	3	1	1	1	1	2	1	1	3	1	2
Rodriquez Portal – 2009^r4^	Prospective case–control	SMRP^(s)^	2	1	1	1	3	2	1	1	1	1	2	1	1	1	1
Shigematsu – 2009^r5^	Case–control	Gene-X^(s)^, THBS-2^(s)^	2	2	2	1	3	2	1	1	1	1	2	1	1	1	1
Amati – 2008^r6^	Prospective case–control	80HdG^(s)^, HGF^(s)^, PDGF*β*^(s)^, SMRP^(s)^, VEGF*β*^(s)^, bFGF^(s)^	2	1	1	1	3	2	1	1	1	2	2	1	1	1	1
Creaney – 2008^r7^	Case–control	MPF^(s)^, SMRP^(s)^, osteopontin^(s)^	2	1	2	1	3	2	1	1	1	2	2	1	1	1	2
Iwahori – 2008^r8^	Case–control	MPF^(s)^, SMRP^(s)^	2	2	2	1	3	2	1	1	1	1	2	1	1	1	1
Pass – 2008^r9^	Case–control	SMRP^(es)^	2	1	2	1	3	2	1	1	1	1	2	1	1	1	2
Schneider – 2008^r10^	Prospective case–control	SMRP^(s)^	2	1	2	1	3	2	3	1	1	1	2	1	3	1	1
Creaney – - 2007^r11 b^	Case–control	CA125^(s)^	2	2	2	1	2	2	1	1	1	1	1	1	1	1	1
Creaney – 2007^r12 c^	Retrospective cohort of consecutive patients with pleural effusion	SMRP^(e)^	2	1	1	1	3	1	1	1	1	1	2	1	1	1	2
Cristaudo – 2007^r13^	Case–control	SMRP^(s)^	2	1	2	1	3	2	1	1	1	1	2	1	1	1	1
Di Serio – 2007^r14^	Case–control	SMRP^(s)^	2	1	2	1	3	2	3	1	1	1	2	1	3	1	1
Grigoriu – 2007^r15 b^	Case–control	Osteopontin^(s)^	2	1	1	1	3	1	1	1	1	2	2	1	3	1	2
Shiomi – 2007^r16^	Prospective case–control	MPF^(s)^	2	1	1	1	3	2	1	1	1	1	1	1	1	1	1
Van den Heuvel – 2007^r17^	Retrospective case–control	CEA^(s)^, CYFRA21-1^(s)^, SMRP^(s)^	2	1	1	1	3	2	1	1	1	1	2	1	1	1	1
Welker – 2007^r18^	Case–control	HA^(e)^	2	2	2	2	3	2	1	1	1	1	1	1	3	1	1
Onda – 2006^r19^	Retrospective case–control	MPF^(s)^	2	1	2	1	3	2	1	1	1	1	2	1	1	1	1
Filiberti – 2005^r20^	Prospective case–control	PDGF-AB^(s)^	2	1	2	1	3	2	1	1	1	1	1	1	1	1	1
Pass – 2005^r21^	Case–control	Osteopontin^(s)^	2	1	1	1	3	2	1	1	1	1	1	1	1	1	1
Scherpereel – 2005^r22^	Prospective cohort of consecutive patients with suspected or recently diagnosed mesothelioma	SMRP^(es)^	1	1	1	1	3	1	1	1	1	1	2	1	3	1	1
Neri – 2003^r23^	Prospective case–control	p53^(s)^	2	1	1	1	3	1	1	1	1	1	2	1	1	1	1
Villena – 2003^r24^	Prospective cohort of patients with pleural effusion	CA15-3^(e)^, CA549^(e)^, CA72-4^(e)^, CEA^(e)^	2	1	1	1	3	3	1	1	1	1	1	1	1	1	1
Creaney - 2001^r25^	Case–control	p53^(s)^	2	2	2	1	3	2	1	1	1	1	2	1	1	1	1
Paganuzzi – 2001^r26^	Cohort of consecutive patients with pleural effusion	CEA^(e)^, CYFRA21-1^(e)^	2	2	2	1	3	1	3	1	1	1	2	1	3	1	1
Fuhrman – 2000^r27^	Prospective case–control	CEA^(es)^, HA^(e)^	2	2	2	1	3	2	1	1	1	1	2	1	1	1	2
Alatas – 1999^r28^	Case–control	CA15-3^(es)^, CA19-9^(e)^, CEA^(es)^, CYFRA21-1^(es)^, NSE^(es)^, TSA^(es)^	2	1	2	1	3	2	1	1	1	1	2	1	1	1	1
Miedouge – 1999^r29^	Retrospective case–control	CA15-3^(e)^, CA19-9^(e)^, CA72-4^(e)^, CEA^(e)^, CYFRA21-1^(e)^, NSE^(e)^, SCC^(e)^	2	2	1	1	3	2	3	1	1	1	1	1	1	1	1
Nisman – 1998^r30^	Case–control	CEA^(s)^, CYFRA21-1^(s)^, TPS^(s)^	2	2	2	1	3	2	1	1	1	1	1	1	1	1	1
Atagi – 1997^r31^	Prospective cohort of consecutive patients with pleural effusion or previously diagnosed mesothelioma	CEA^(e)^, HA^(e)^	2	2	1	1	3	1	3	3	1	1	1	1	3	1	2
Ebert – 1997^r32^	Prospective case–control	CEA^(s)^, CYFRA21-1^(s)^, NSE^(s)^, TPA-M^(s)^, TPS^(s)^	2	2	2	1	3	2	1	1	1	1	1	1	1	1	2
Shijubo – 1995^r33^	Case–control	CEA^(e)^, SP-A^(e)^	2	2	2	1	3	2	1	1	1	1	2	1	1	1	1
Villena – 1995^r34^	Prospective cohort of patients with pleural effusion	CA15-3^(e)^, CA19-9^(e)^, CA72-4^(e)^, CEA^(e)^	2	1	1	1	3	3	1	1	1	1	2	1	1	1	1
Whitaker – 1986^r35^	Retrospective case–control	CEA^(e)^	2	2	2	1	3	2	1	1	1	1	1	1	1	1	1
Fravelli – 1984^r36^	Cohort of patients with pleural effusion	CEA^(e)^	2	1	2	1	3	3	1	1	1	1	1	1	1	1	1
																	
*Studying immunohistochemical markers*																	
Shen – 2009^r37^	Retrospective case–control	EMA, Glut-1m, Glut-1p, XIAP	2	2	2	1	3	2	3	3	1	2	2	1	1	3	1
Slipicevic – 2009^r38^	Case–control	IGF-II, IGFBP3	2	2	2	1	3	2	3	3	1	2	2	3	3	3	1
Yuan – 2009^r39^	Case–control	B72-3, Ber-EP4, EMA, Tenascin-X, calretinin	2	2	2	1	3	2	1	1	1	2	2	3	1	3	1
Bhalla – 2007^r40^	Retrospective case–control	CK5, D2-40, calretinin, podoplanin	2	2	2	1	3	2	1	1	1	2	2	3	1	3	1
Facchetti – 2007^r41^	Retrospective cohort of patients with effusion	Claudin4	2	2	2	1	3	2	1	1	1	1	1	3	1	3	1
Grefte – 2007^r42^	Retrospective case–control	B72-3, Ber-EP4, CEA, EMA, HMFG-2, calretinin	2	2	2	1	3	2	3	3	1	2	2	1	3	2	1
Kleinberg – 2007^r43^	Retrospective case–control	Claudin1, claudin3	2	2	2	1	3	2	1	1	1	2	2	3	1	3	1
Pu – 2007^r44^	Retrospective case–control	MOC-31, WT-1, mesothelin, p63	2	2	2	1	3	2	1	1	1	2	2	1	1	3	2
Shield – 2007^r45^	Retrospective case–control	CK5/6, calretinin	2	2	2	1	3	2	2	3	1	2	1	3	2	3	1
Aerts – 2006^r46^	Prospective case–control	Ber-EP4, CEA, EMA, TAG-72	2	2	2	1	3	2	3	3	1	2	2	1	3	2	1
Bassarova – 2006^r47^	Case–control	D2-40	2	2	2	1	3	2	3	3	1	2	2	3	3	3	1
Li – 2006^r48^	Retrospective case–control	Ber-EP4, CAM5-2, CEA, CK5/6, K903, calretinin	2	2	2	1	3	2	3	3	1	2	2	3	3	3	1
Saad – 2006^r49^	Retrospective case–control	CK5/6, D2-40, TTF-1, WT-1, calretinin, p63	2	2	2	1	3	2	3	1	1	2	1	1	3	2	1
Sivertsen – 2006^r50^	Case-control	E-cadherin, N-cadherin, P-cadherin	2	2	2	1	3	2	3	3	2	2	2	3	3	3	1
Afify – 2005^r51^	Retrospective case–control	CD44S, HA	2	2	2	1	3	2	1	1	1	2	1	3	1	3	1
Hecht – 2005^r52^	Retrospective case–control	MOC-31	2	2	2	2	3	2	1	1	1	2	2	3	1	3	1
Saad – 2005^r53^	Retrospective case–control	EMA	2	2	2	1	3	2	1	1	1	1	2	1	1	2	1
Saqi – 2005^r54^	Case–control	CD138	2	2	2	1	3	2	1	1	1	1	2	3	1	3	1
Schonherr – 2004^r55^	Case–control	Ki67	2	2	2	1	3	2	1	1	1	1	1	3	1	3	1
Afify – 2002^r56^	Retrospective case–control	TTF-1	2	2	2	1	3	2	1	1	1	2	1	3	1	3	1
Afify – 2002^r57^	Retrospective case–control	Actin, desmin, myogenin, myoglobin	2	2	2	1	3	2	1	1	1	2	1	3	1	3	1
Davidson – 2001^r58 b^	Case–control	CA125, CEA, desmin, p53, vimentin	2	2	2	1	3	2	1	2	1	2	1	1	2	1	1
Hecht – 2001^r59^	Retrospective case–control	WT1	2	2	2	1	3	2	3	3	1	2	2	3	3	3	1
Hecht – 2001^r60^	Retrospective case–control	TTF-1	2	2	2	1	3	2	1	1	1	2	2	3	1	3	1
Simsir – 2001^r61^	Retrospective case–control	SV-40	2	2	2	3	2	2	1	1	1	2	2	3	1	3	1
Wieczorek – 2000^r62^	Retrospective case–control	Calretinin	2	2	2	1	3	2	1	1	1	1	1	1	1	2	1
Dejmek – 1999^r63^	Retrospective case–control	CAM5-2, Leu-M1	2	2	2	1	3	2	2	3	1	2	2	3	3	3	2
Motherby – 1999^r64^	Retrospective cohort of patient with pleural effusion	Ber-EP4, CEA, EMA, Leu-M1	2	2	2	3	3	2	2	2	1	2	2	3	2	3	2
Simir – 1999^r65^	Retrospective case–control	E-cadherin, N-cadherin, calretinin	2	2	2	3	3	2	1	1	1	2	2	3	1	3	1
Ascoli – 1997^r66^	Case–control	HBME-1	2	2	2	1	3	2	2	3	1	2	1	3	3	3	1
Delahaye – 1997^r67^	Case–control	B72-3, Ber-EP4, CEA, Leu-M1, MOC-31	2	2	2	1	3	2	3	3	1	2	2	3	3	3	1
Ascoli – 1995^r68^	Case–control	Ber-EP4, EMA, cytokeratin, thrombomodulin	2	2	2	1	3	2	2	3	1	2	1	3	3	3	1
Baars – 1994^r69^	Retrospective case–control	Keratin7	2	2	2	1	3	2	1	1	1	2	2	3	1	3	1
Donna – 1992^r70^	Case–control	Mesothelin	2	2	2	1	3	2	2	1	1	1	2	3	3	3	1
Betta – 1991^r71^	Case–control	B72-3	2	2	2	1	3	2	3	1	1	2	2	3	3	3	1
Delahaye – 1991^r72 b^	Retrospective case–control	EMA, OV632	2	2	2	1	3	2	1	1	1	2	2	3	1	3	1
Kuhlman – 1991^r73^	Retrospective case–control	B72-3, BMA-120, Ber-EP4, CEA, HEA-125, cytokeratin, vimentin	2	2	2	1	3	2	1	1	1	2	2	3	1	3	1
Linari – 1989^r74^	Prospective case–control	HMFG-2	2	2	2	3	3	2	2	3	1	2	2	3	2	3	1
Cibas – 1987^r75^	Retrospective case–control	CEA, EMA, HMFG-2, keratin	2	1	2	1	3	2	1	1	1	2	2	3	1	3	1
Ghosh – 1987^r76^	Retrospective cohort of patients with suspected/diagnosed mesothelioma	CA1/2, CEA, HMFG-2	1	2	2	1	3	2	1	1	1	2	2	3	1	3	1
Walts – 1983^r77^	Retrospective case–control	Keratin	2	2	2	1	3	2	3	1	1	2	1	3	3	3	1
																	
*Studying genetic markers*																	
Illei – 2003^r78^	Prospective case–control	CDKN2A-deletion^(e)^	2	1	2	1	3	2	3	1	1	1	2	3	3	3	1
Flores-Staino – 2009^r79^	Case–control	CDKN2A-deletion^(e)^	2	2	2	1	3	2	1	1	1	1	2	3	1	3	1
																	
*Studying several types of markers*																	
Botelho – 2008^r80^	Retrospective case–control	Genetic: CDKN2A-deletion^(e)^ immunohistochemical: Ber-EP4, calretinin	2	2	2	2	3	2	2	1	1	2	2	3	2	3	1
Creaney – 2008^r81^	Retrospective case–control	Biomarkers: CA15-3^(es)^ immunohistochemical: EMA	2	2	2	1	3	2	3	3	1	1	2	3	3	3	2
Dejmek – 2005^r82^	Case–control	Biomarkers: HA^(e)^ immunohistochemical: Ber-EP4, CEA, Ca125, EMA, HBME-1, Sial-Tn, thrombomodulin, vimentin	2	2	2	1	3	2	2	1	1	1	2	3	3	3	2

^(e)^, assessed in effusion; ^(s)^, assessed in serum; ^(es)^, assessed in effusion and serum.

a See Supplementary Appendix for criteria on quality assessment, items were scored 1=yes, 2=no, 3=unclear.

b Also studied other markers that we did not incorporate because of overlap with other studies.

c Also studied SMRP in serum that we did not incorporate because of overlap with other studies.
