# Supplementary material for: Markers for the non-invasive diagnosis of mesothelioma: a systematic review
Source: Br J Cancer. 2011 Mar 29;104(8):1325–33. doi: 10.1038/bjc.2011.104 (PMC3078590; doi:10.1038/bjc.2011.104)
Supplement: Supplementary Appendix Tables [file bjc2011104x1.doc]

**Appendices (online only)**

***Appendix 1. The search strategy***

| **Database** | **Search strategy** |
| --- | --- |
| Pubmed | ((mesothelioma[mesh] OR mesothelioma*[all]) AND ("Body Fluids"[Mesh] OR pleural effusion[Mesh] OR pericardial effusion[Mesh] OR cytology[tiab] OR cytologic*[tiab] OR serum[All] OR blood[all] OR serous[all] OR effusion[all] OR effusions[all] OR fluid*[all] OR ascites[all]) AND (biomarker[Mesh] OR biomarker*[all] OR marker*[all] OR protein[all] OR peptide[all] OR antibody[all] OR elisa[all] OR gene[all] OR genetic*[all] OR oncogene*[all] OR chromosome[all] OR chromosomal[all])) OR ((mesothelioma[mesh] OR mesothelioma*[all]) AND (immunocytochemistry[tiab] OR immunocytology[tiab])) |
| Embase | ((mesothelioma* OR 'mesothelioma'/exp) AND [embase]/lim AND ('biomarker'/exp OR biomarker* OR marker* OR protein OR peptide OR antibody OR elisa OR gene OR genetic* OR oncogene* OR chromosome OR chromosomal) AND ('body fluids'/exp OR 'pleural effusion'/exp OR 'pericardial effusion' OR cytology:ab,ti OR cytologic*:ab,ti OR serum OR blood OR serous OR effusion OR effusions OR fluid* OR ascites)) OR ((mesothelioma* OR 'mesothelioma'/exp) AND [embase]/lim AND (immunocytochemistry:ab,ti OR immunocytology:ab,ti)) |

***Appendix 2. The Quadas instrument to assess methodological quality of individual diagnostic accuracy studies***

Study quality was assessed using the QUADAS criteria[Whiting et al., 2003], with each item scored “yes”, “no”, or “unclear”. The items of the QUADAS tool and their interpretation are described below. In the definitions, the index test refers to the marker under study (i.e. a serum marker, effusion marker, immunohistochemical marker or genetic marker).

*1a. Representative spectrum?*

The participants of interest were patients with suspected mesothelioma, selected following a prospective, consecutive patient inclusion. Retrospective cohort studies and case-control studies were scored as no.

*1b. Clear description of the study participants?*

The description of the study participants was considered sufficient if the age distribution, female-to-male ratio and description of the disease were sufficiently described (per study group). If not, this item was scored as no. This item was supplementary to the first QUADAS criterion as a description of the characteristics is important to judge the population actually included and therefore to judge generalisability.

*2. Clear description of selection criteria?*

The description of the selection criteria was considered sufficient when time period and location of recruitment and setting were described, if it was clear if data collection was planned before (prospective study) or after (retrospective study) the index test and reference standard were performed, and how participants were recruited, i.e. based on presented symptoms or on the fact that the participants had received the index tests or a (specific) reference standard.

*3. Is the reference standard likely to correctly classify the target condition?*

This item was scored yes when diagnosis of mesothelioma was based on at least cytology or histology.

*4. Is the time period between reference standard and index test short enough to be reasonably sure that the target condition did not change between the two tests?*

This item was scored yes when the time interval between index test and reference test was less than one month in >80% of the mesothelioma patients. Unclear was utilized when this percentage could not be calculated or no information was given. When it was stated that specimens were collected at time of diagnosis without further specification this item was scored unclear as well.

*5. Did the whole sample or a random selection of the sample, receive verification using a reference standard of diagnosis?*

If all patients or a random selection of patients received verification with the reference standard then the item was scored yes, even if the reference standard was not the same (see next item) for all patients. When no information was provided about the flow of the patients, or patients were not selected consecutively, then this item was scored unclear. Case-control or retrospective cohort studies scored no on this item as this design commonly leads to partial verification bias due to a non-random selection.[Whiting et al., 2004;Rutjes et al., 2005;Biesheuvel et al., 2008;Mol et al., 2003;Lijmer et al., 1999;Begg and Greenes, 1983]

*6. Did patients receive the same reference standard regardless of the index test result?*

This item was scored yes when patients received the same reference standard or when the index test was performed after the reference test (as it is unlikely that the reference test will affect the performance of the index test). Unclear was used when it was uncertain if the index test was (also) performed before or after the reference test.

*7. Was the reference standard independent of the index test (i.e. the index test did not form part of the reference standard)?*

When the results of the index test were not incorporated in the final diagnosis of all study patients the item was scored yes, or when the index test was performed after the established diagnosis. When evaluating cytologic markers and no details were provided on the cytologic markers that were used in the cytology reference standard the item was scored unclear.

*8a. Was the execution of the index test described in sufficient detail to permit replication of the test?*

To score yes required that the description included how antibodies or markers of genes were retrieved (or the name of the manufacturer) and type of detection system. In addition, the process of handling and preparation of samples (if cell blocks, cytospins, smears, fresh or stored samples were used) had to be included in case of immunohistochemical markers.

*8b.Was the cut-off value described clearly?*

This item was scored yes when a clear definition of units, cut-off point or categories of the results of the index test were provided. For immunohistochemical markers this item was scored yes when it was clear which percentage of cells, type of staining pattern and intensity were considered as positive.

*9. Was the execution of the reference standard described in sufficient detail to permit its replication?*

To score yes the description had to include the criteria that was used in the reference standard of mesothelioma (i.e. based on morphologic features, certain cytological or histological markers (including details on type) or electron microscope). When it was stated that diagnosis was based following published guidelines this item was scored as yes. At first, to score yes the number of mesothelioma patients that was diagnosed by either cytology or histology had to be reported, however this was almost not reported in the studies and therefore not incorporated.

*10. Were the index test results interpreted without knowledge of the results of the reference standard?*

To confirm that this blinding was accounted for, a clear statement in the text such as “personnel/observers who performed the biomarker assessment were blinded/unaware of the patient’s diagnosis” had to be given. If there was a statement that blinding was not accounted for the item was scored as no. If no statement on blinding was given the item was scored unclear. If the index test was entirely quantitative (and required no subjective interpretation), e.g. a test using ELISA or immunoradiometric assay, then this item was scored yes.

*11. Were the reference standard results interpreted without knowledge of the results of the index test?*

To confirm that this blinding was accounted for, a clear statement in the text such as “personnel/observers who performed the biomarker assessment were blinded/unaware of the results of the index test” had to be given. If it was clear that blinding was not accounted for the item was scored as no. If no statement on blinding was given and the index test did not form part of the reference standard the item was scored unclear. When it was clear that the index test was performed subsequent to the diagnosis we scored this item as yes. If it was unclear whether the index was (also) performed before or after the reference standard the item was scored as unclear.

*12. Were the same clinical data available when test results were interpreted as would be available when the test is used in practice?*

When it was clear that pre-test or other clinical data were available when the index test (biomarker assay) was interpreted, then the item was scored yes. When it was stated that observers of the biomarker assay were blinded to clinical data the item was scored with no. Unclear was used when no statement on the availability of pre-test or clinical data was provided. If the test was entirely objective (i.e. a test using ELISA or immunoradiometric assay) then this item was scored yes.

*13. Were uninterpretable/ intermediate/ test results reported or other missing test results explained?*

If uninterpretable, failed or intermediate results were documented or all results were available for all patients who entered the study then the item was scored yes. If it was apparent that results were missing but no explanation was given, the item was scored no. When missing results were due to the fact that specimens were not available for all participants and it was not further specified why, we scored this item as no.

***Appendix 3. Reference list of studies included in the systematic review***

r1 Aleman C, Manuel PJ, Segura MA, Alegre J, Esquerda A, Ruiz E et al. Pleural fluid mesothelin for the differential diagnosis of exudative pleural effusions. Med Clin (Barc ) 2009 October 3;133(12):449-53.

r2 Davies HE, Sadler RS, Bielsa S, Maskell NA, Rahman NM, Davies RJ et al. The Clinical Impact and Reliability of Pleural Fluid Mesothelin in Undiagnosed Pleural Effusions. Am J Respir Crit Care Med 2009 March 19.

r3 Grigoriu B, Chahine B, Zerimech F, Gregoire M, Balduyck M, Copin MC et al. Serum mesothelin has a higher diagnostic utility than hyaluronic acid in malignant mesothelioma. Clin Biochem 2009 July;42(10-11):1046-50.

r4 Rodriguez Portal JA, Rodriguez BE, Rodriguez RD, Alfageme M, I, Quero MA, Diego RC et al. Serum levels of soluble mesothelin-related peptides in malignant and nonmalignant asbestos-related pleural disease: relation with past asbestos exposure. Cancer Epidemiol Biomarkers Prev 2009 February;18(2):646-50.

r5 Shigematsu Y, Hanagiri T, Kuroda K, Baba T, Mizukami M, Ichiki Y et al. Malignant mesothelioma-associated antigens recognized by tumor-infiltrating B cells and the clinical significance of the antibody titers. Cancer Sci 2009 April 30.

r6 Amati M, Tomasetti M, Scartozzi M, Mariotti L, Alleva R, Pignotti E et al. Profiling tumor-associated markers for early detection of malignant mesothelioma: an epidemiologic study. Cancer Epidemiol Biomarkers Prev 2008 January;17(1):163-70.

r7 Creaney J, Yeoman D, Demelker Y, Segal A, Musk AW, Skates SJ et al. Comparison of osteopontin, megakaryocyte potentiating factor, and mesothelin proteins as markers in the serum of patients with malignant mesothelioma. J Thorac Oncol 2008 August;3(8):851-7.

r8 Iwahori K, Osaki T, Serada S, Fujimoto M, Suzuki H, Kishi Y et al. Megakaryocyte potentiating factor as a tumor marker of malignant pleural mesothelioma: evaluation in comparison with mesothelin. Lung Cancer 2008 October;62(1):45-54.

r9 Pass HI, Wali A, Tang N, Ivanova A, Ivanov S, Harbut M et al. Soluble mesothelin-related peptide level elevation in mesothelioma serum and pleural effusions. Ann Thorac Surg 2008 January;85(1):265-72.

r10 Schneider J, Hoffmann H, Dienemann H, Herth FJ, Meister M, Muley T. Diagnostic and prognostic value of soluble mesothelin-related proteins in patients with malignant pleural mesothelioma in comparison with benign asbestosis and lung cancer. J Thorac Oncol 2008 November;3(11):1317-24.

r11 Creaney J, van B, I, Hof M, Segal A, Musk AW, de KN et al. Combined CA125 and mesothelin levels for the diagnosis of malignant mesothelioma. Chest 2007 October;132(4):1239-46.

r12 Creaney J, Yeoman D, Naumoff LK, Hof M, Segal A, Musk AW et al. Soluble mesothelin in effusions: a useful tool for the diagnosis of malignant mesothelioma. Thorax 2007 July;62(7):569-76.

r13 Cristaudo A, Foddis R, Vivaldi A, Guglielmi G, Dipalma N, Filiberti R et al. Clinical significance of serum mesothelin in patients with mesothelioma and lung cancer. Clin Cancer Res 2007 September 1;13(17):5076-81.

r14 Di SF, Fontana A, Loizzi M, Capotorto G, Maggiolini P, Mera E et al. Mesothelin family proteins and diagnosis of mesothelioma: analytical evaluation of an automated immunoassay and preliminary clinical results. Clin Chem Lab Med 2007;45(5):634-8.

r15 Grigoriu BD, Scherpereel A, Devos P, Chahine B, Letourneux M, Lebailly P et al. Utility of osteopontin and serum mesothelin in malignant pleural mesothelioma diagnosis and prognosis assessment. Clin Cancer Res 2007 May 15;13(10):2928-35.

r16 Shiomi K, Hagiwara Y, Sonoue K, Segawa T, Miyashita K, Maeda M et al. Sensitive and specific new enzyme-linked immunosorbent assay for N-ERC/mesothelin increases its potential as a useful serum tumor marker for mesothelioma. Clin Cancer Res 2008 March 1;14(5):1431-7.

r17 van den Heuvel MM, Korse CM, Bonfrer JM, Baas P. Non-invasive diagnosis of pleural malignancies: the role of tumour markers. Lung Cancer 2008 March;59(3):350-4.

r18 Welker L, Muller M, Holz O, Vollmer E, Magnussen H, Jorres RA. Cytological diagnosis of malignant mesothelioma - Improvement by additional analysis of hyaluronic acid in pleural effusions. Virchows Arch 2007;450(4):455-61.

r19 Onda M, Nagata S, Ho M, Bera TK, Hassan R, Alexander RH et al. Megakaryocyte potentiation factor cleaved from mesothelin precursor is a useful tumor marker in the serum of patients with mesothelioma. Clin Cancer Res 2006 July 15;12(14 Pt 1):4225-31.

r20 Filiberti R, Marroni P, Neri M, Ardizzoni A, Betta PG, Cafferata MA et al. Serum PDGF-AB in pleural mesothelioma. Tumour Biol 2005 September;26(5):221-6.

r21 Pass HI, Lott D, Lonardo F, Harbut M, Liu Z, Tang N et al. Asbestos exposure, pleural mesothelioma, and serum osteopontin levels. N Engl J Med 2005 October 13;353(15):1564-73.

r22 Scherpereel A, Grigoriu B, Conti M, Gey T, Gregoire M, Copin MC et al. Soluble mesothelin-related peptides in the diagnosis of malignant pleural mesothelioma. Am J Respir Crit Care Med 2006 May 15;173(10):1155-60.

r23 Neri M, Betta P, Marroni P, Filiberti R, Cafferata M, Mereu C et al. Serum anti-p53 autoantibodies in pleural malignant mesothelioma, lung cancer and non-neoplastic lung diseases. Lung Cancer 2003 February;39(2):165-72.

r24 Villena V, Lopez-Encuentra A, Echave-Sustaeta J, Martin-Escribano P, Ortuno-de-Solo B, Estenoz-Alfaro J. Diagnostic value of CA 549 in pleural fluid. Comparison with CEA, CA 15.3 and CA 72.4. Lung Cancer 2003 June;40(3):289-94.

r25 Creaney J, McLaren BM, Stevenson S, Musk AW, de KN, Robinson BW et al. p53 autoantibodies in patients with malignant mesothelioma: stability through disease progression. Br J Cancer 2001 January 5;84(1):52-6.

r26 Paganuzzi M, Onetto M, Marroni P, Filiberti R, Tassara E, Parodi S et al. Diagnostic value of CYFRA 21-1 tumor marker and CEA in pleural effusion due to mesothelioma. Chest 2001 April;119(4):1138-42.

r27 Fuhrman C, Duche JC, Chouaid C, Abd A, I, Atassi K, Monnet I et al. Use of tumor markers for differential diagnosis of mesothelioma and secondary pleural malignancies. Clin Biochem 2000 July;33(5):405-10.

r28 Alatas F, Alatas O, Metintas M, Colak O, Harmanci E, Demir S. Diagnostic value of CEA, CA 15-3, CA 19-9, CYFRA 21-1, NSE and TSA assay in pleural effusions. Lung Cancer 2001 January;31(1):9-16.

r29 Miedouge M, Rouzaud P, Salama G, Pujazon MC, Vincent C, Mauduyt MA et al. Evaluation of seven tumour markers in pleural fluid for the diagnosis of malignant effusions. Br J Cancer 1999 November;81(6):1059-65.

r30 Nisman B, Barak V, Heching N, Kramer M, Reinus C, Lafair J. Cytokeratin markers in malignant pleural mesothelioma. Cancer Detect Prev 1998;22(5):416-21.

r31 Atagi S, Ogawara M, Kawahara M, Sakatani M, Furuse K, Ueda E et al. Utility of hyaluronic acid in pleural fluid for differential diagnosis of pleural effusions: likelihood ratios for malignant mesothelioma. Jpn J Clin Oncol 1997 October;27(5):293-7.

r32 Ebert W, Hoppe M, Muley T, Drings P. Monitoring of therapy in inoperable lung cancer patients by measurement of CYFRA 21-1, TPA- TP CEA, and NSE. Anticancer Res 1997 July;17(4B):2875-8.

r33 Shijubo N, Honda Y, Fujishima T, Takahashi H, Kodama T, Kuroki Y et al. Lung surfactant protein-A and carcinoembryonic antigen in pleural effusions due to lung adenocarcinoma and malignant mesothelioma. Eur Respir J 1995 March;8(3):403-6.

r34 Villena V, Lopez-Encuentra A, Echave-Sustaeta J, Martin-Escribano P, Ortuno-de-Solo B, Estenoz-Alfaro J. Diagnostic value of CA 72-4, carcinoembryonic antigen, CA 15-3, and CA 19-9 assay in pleural fluid. A study of 207 patients. Cancer 1996 August 15;78(4):736-40.

r35 Whitaker D, Shilkin KB, Stuckey M, Nieuwhof WN. Pleural fluid CEA levels in the diagnosis of malignant mesothelioma. Pathology 1986 July;18(3):328-9.

r36 Faravelli B, D'Amore E, Nosenzo M, Betta PG, Donna A. Carcinoembryonic antigen in pleural effusions. Diagnostic value in malignant mesothelioma. Cancer 1984 March 1;53(5):1194-7.

r37 Shen J, Pinkus GS, Deshpande V, Cibas ES. Usefulness of EMA, GLUT-1, and XIAP for the cytologic diagnosis of malignant mesothelioma in body cavity fluids. Am J Clin Pathol 2009 April;131(4):516-23.

r38 Slipicevic A, Oy GF, Askildt IC, Holth A, Hellesylt E, Florenes VA et al. Diagnostic and prognostic role of the insulin growth factor pathway members insulin-like growth factor-II and insulin-like growth factor binding protein-3 in serous effusions. Hum Pathol 2009 April;40(4):527-37.

r39 Yuan Y, Nymoen DA, Stavnes HT, Rosnes AK, Bjorang O, Wu C et al. Tenascin-X is a novel diagnostic marker of malignant mesothelioma. Am J Surg Pathol 2009 November;33(11):1673-82.

r40 Bhalla R, Siddiqui MT, Mandich D, Cartun RW, Fiel-Gan MD, Nassar A et al. Diagnostic utility of D2-40 and podoplanin in effusion cell blocks. Diagn Cytopathol 2007 June;35(6):342-7.

r41 Facchetti F, Lonardi S, Gentili F, Bercich L, Falchetti M, Tardanico R et al. Claudin 4 identifies a wide spectrum of epithelial neoplasms and represents a very useful marker for carcinoma versus mesothelioma diagnosis in pleural and peritoneal biopsies and effusions. Virchows Arch 2007 September;451(3):669-80.

r42 Grefte JM, de Wilde PC, Salet-van de Pol MR, Tomassen M, Raaymakers-van Geloof WL, Bulten J. Improved identification of malignant cells in serous effusions using a small, robust panel of antibodies on paraffin-embedded cell suspensions. Acta Cytol 2008 January;52(1):35-44.

r43 Kleinberg L, Holth A, Fridman E, Schwartz I, Shih I, Davidson B. The diagnostic role of claudins in serous effusions. Am J Clin Pathol 2007 June;127(6):928-37.

r44 Pu RT, Pang Y, Michael CW. Utility of WT-1, p63, MOC31, mesothelin, and cytokeratin (K903 and CK5/6) immunostains in differentiating adenocarcinoma, squamous cell carcinoma, and malignant mesothelioma in effusions. Diagn Cytopathol 2008 January;36(1):20-5.

r45 Shield PW, Koivurinne K. The value of calretinin and cytokeratin 5/6 as markers for mesothelioma in cell block preparations of serous effusions. Cytopathology 2008 August;19(4):218-23.

r46 Aerts JG, Delahaye M, van der Kwast TH, Davidson B, Hoogsteden HC, van Meerbeeck JP. The high post-test probability of a cytological examination renders further investigations to establish a diagnosis of epithelial malignant pleural mesothelioma redundant. Diagn Cytopathol 2006 August;34(8):523-7.

r47 Bassarova AV, Nesland JM, Davidson B. D2-40 is not a specific marker for cells of mesothelial origin in serous effusions. Am J Surg Pathol 2006 July;30(7):878-82.

r48 Li Q, Bavikatty N, Michael CW. The role of immunohistochemistry in distinguishing squamous cell carcinoma from mesothelioma and adenocarcinoma in pleural effusion. Semin Diagn Pathol 2006 February;23(1):15-9.

r49 Saad RS, Lindner JL, Lin X, Liu YL, Silverman JF. The diagnostic utility of D2-40 for malignant mesothelioma versus pulmonary carcinoma with pleural involvement. Diagn Cytopathol 2006 December;34(12):801-6.

r50 Sivertsen S, Berner A, Michael CW, Bedrossian C, Davidson B. Cadherin expression in ovarian carcinoma and malignant mesothelioma cell effusions. Acta Cytol 2006 November;50(6):603-7.

r51 Afify AM, Stern R, Michael CW. Differentiation of mesothelioma from adenocarcinoma in serous effusions: the role of hyaluronic acid and CD44 localization. Diagn Cytopathol 2005 March;32(3):145-50.

r52 Hecht JL, Pinkus JL, Pinkus GS. Monoclonal antibody MOC-31 reactivity as a marker for adenocarcinoma in cytologic preparations. Cancer 2006 February 25;108(1):56-9.

r53 Saad RS, Cho P, Liu YL, Silverman JF. The value of epithelial membrane antigen expression in separating benign mesothelial proliferation from malignant mesothelioma: a comparative study. Diagn Cytopathol 2005 March;32(3):156-9.

r54 Saqi A, Yun SS, Yu GH, Alexis D, Taub RN, Powell CA et al. Utility of CD138 (syndecan-1) in distinguishing carcinomas from mesotheliomas. Diagn Cytopathol 2005 August;33(2):65-70.

r55 Schonherr A, Bayer M, Bocking A. Diagnostic and prognostic value of Ki67 proliferation fraction in serous effusions. Cell Oncol 2004;26(1-2):57-62.

r56 Afify AM, al-Khafaji BM. Diagnostic utility of thyroid transcription factor-1 expression in adenocarcinomas presenting in serous fluids. Acta Cytol 2002 July;46(4):675-8.

r57 Afify AM, al-Khafaji BM, Paulino AF, Davila RM. Diagnostic use of muscle markers in the cytologic evaluation of serous fluids. Appl Immunohistochem Mol Morphol 2002 June;10(2):178-82.

r58 Davidson B, Nielsen S, Christensen J, Asschenfeldt P, Berner A, Risberg B et al. The role of desmin and N-cadherin in effusion cytology: a comparative study using established markers of mesothelial and epithelial cells. Am J Surg Pathol 2001 November;25(11):1405-12.

r59 Hecht JL, Lee BH, Pinkus JL, Pinkus GS. The value of Wilms tumor susceptibility gene 1 in cytologic preparations as a marker for malignant mesothelioma. Cancer 2002 April 25;96(2):105-9.

r60 Hecht JL, Pinkus JL, Weinstein LJ, Pinkus GS. The value of thyroid transcription factor-1 in cytologic preparations as a marker for metastatic adenocarcinoma of lung origin. Am J Clin Pathol 2001 October;116(4):483-8.

r61 Simsir A, Fetsch P, Bedrossian CW, Ioffe OB, Abati A. Absence of SV-40 large T antigen (Tag) in malignant mesothelioma effusions: an immunocytochemical study. Diagn Cytopathol 2001 October;25(4):203-7.

r62 Wieczorek TJ, Krane JF. Diagnostic utility of calretinin immunohistochemistry in cytologic cell block preparations. Cancer 2000 October 25;90(5):312-9.

r63 Dejmek A, Hjerpe A. Reactivity of six antibodies in effusions of mesothelioma, adenocarcinoma and mesotheliosis: stepwise logistic regression analysis. Cytopathology 2000 February;11(1):8-17.

r64 Motherby H, Kube M, Friedrichs N, Nadjari B, Knops K, Donner A et al. Immunocytochemistry and DNA-image cytometry in diagnostic effusion cytology I. Prevalence of markers in tumour cell positive and negative smears. Anal Cell Pathol 1999;19(1):7-20.

r65 Simsir A, Fetsch P, Mehta D, Zakowski M, Abati A. E-cadherin, N-cadherin, and calretinin in pleural effusions: the good, the bad, the worthless. Diagn Cytopathol 1999 March;20(3):125-30.

r66 Ascoli V, Carnovale-Scalzo C, Taccogna S, Nardi F. Utility of HBME-1 immunostaining in serous effusions. Cytopathology 1997 October;8(5):328-35.

r67 Delahaye M, van der HF, van der Kwast TH. Complementary value of five carcinoma markers for the diagnosis of malignant mesothelioma, adenocarcinoma metastasis, and reactive mesothelium in serous effusions. Diagn Cytopathol 1997 August;17(2):115-20.

r68 Ascoli V, Scalzo CC, Taccogna S, Nardi F. The diagnostic value of thrombomodulin immunolocalization in serous effusions. Arch Pathol Lab Med 1995 December;119(12):1136-40.

r69 Baars JH, De Ruijter JLM, Smedts F, Van Niekerk CC, Poels LG, Seldenrijk CA et al. The applicability of a keratin 7 monoclonal antibody in routinely papanicolaou-stained cytologic specimens for the differential diagnosis of carcinomas. Am J Clin Pathol 1994;101(3):257-61.

r70 Donna A, Betta PG, Bellingeri D, Tallarida F, Pavesi M, Pastormerlo M. Cytologic diagnosis of malignant mesothelioma in serous effusions using an antimesothelial-cell antibody. Diagn Cytopathol 1992;8(4):361-5.

r71 Betta PG, Pavesi M, Pastormerlo M, Tallarida F, Bellingeri D, Bocca R. Use of monoclonal antibody B72.3 as a marker of metastatic carcinoma cells in neoplastic effusions. Pathologica 1991 January;83(1083):99-104.

r72 Delahaye M, Hoogsteden HC, van der Kwast TH. Immunocytochemistry of malignant mesothelioma: OV632 as a marker of malignant mesothelioma. J Pathol 1991 October;165(2):137-43.

r73 Kuhlmann L, Berghauser KH, Schaffer R. Distinction of mesothelioma from carcinoma in pleural effusions. An immunocytochemical study on routinely processed cytoblock preparations. Pathol Res Pract 1991 May;187(4):467-71.

r74 Linari A, Bussolati G. Evaluation of impact of immunocytochemical techniques in cytological diagnosis of neoplastic effusions. J Clin Pathol 1989 November;42(11):1184-9.

r75 Cibas ES, Corson JM, Pinkus GS. The distinction of adenocarcinoma from malignant mesothelioma in cell blocks of effusions: the role of routine mucin histochemistry and immunohistochemical assessment of carcinoembryonic antigen, keratin proteins, epithelial membrane antigen, and milk fat globule-derived antigen. Hum Pathol 1987 January;18(1):67-74.

r76 Ghosh AK, Butler EB. Immunocytological staining reactions of anti-carcinoembryonic antigen, Ca, and anti-human milk fat globule monoclonal antibodies on benign and malignant exfoliated mesothelial cells. J Clin Pathol 1987 December;40(12):1424-7.

r77 Walts AE, Said JW, Shintaku IP, Sassoon AF, Banks-Schlegel S. Keratins of different molecular weight in exfoliated mesothelial and adenocarcinoma cells--an aid to cell identification. Am J Clin Pathol 1984 April;81(4):442-6.

r78 Illei PB, Ladanyi M, Rusch VW, Zakowski MF. The use of CDKN2A deletion as a diagnostic marker for malignant mesothelioma in body cavity effusions. Cancer 2003 February 25;99(1):51-6.

r79 Flores-Staino C, rai-Ramqvist E, Dobra K, Hjerpe A. Adaptation of a commercial fluorescent in situ hybridization test to the diagnosis of malignant cells in effusions. Lung Cancer 2009 June 10.

r80 Onofre FB, Onofre AS, Pomjanski N, Buckstegge B, Grote HJ, Bocking A. 9p21 Deletion in the diagnosis of malignant mesothelioma in serous effusions additional to immunocytochemistry, DNA-ICM, and AgNOR analysis. Cancer 2008 June 25;114(3):204-15.

r81 Creaney J, Segal A, Sterrett G, Platten MA, Baker E, Murch AR et al. Overexpression and altered glycosylation of MUC1 in malignant mesothelioma. Br J Cancer 2008 May 6;98(9):1562-9.

r82 Dejmek A, Hjerpe A. The combination of CEA, EMA, and BerEp4 and hyaluronan analysis specifically identifies 79% of all histologically verified mesotheliomas causing an effusion. Diagn Cytopathol 2005 March;32(3):160-6.

***Appendix 4. Sensitivity and specificity of markers***

Table 2.1: Reported sensitivity and specificity of serum markers per study, stratified by type of marker

| **No.** | **Marker** | **1st Author-year** | **Type of mesthelioma** | **in comparison with** | | | | | |
| --- | --- | --- | --- | --- | --- | --- | --- | --- | --- |
|  |  |  |  | **Malignancy** | | **Non-malignancy** | | **Malignancy and non-malignancy combined** | |
|  |  |  |  | **Sens %**  **TP/FN** | **Spec %**  **(TN/FP)** | **Sens %**  **TP/FN** | **Spec %**  **(TN/FP)** | **Sens %**  **TP/FN** | **Spec %**  **(TN/FP)** |
| 1.1 | SMRP | Rodriquez Portal - 2009^r4^ | pleural |  |  | 72 (26/10) | 72 (235/91) |  |  |
| 1.2 | SMRP | Schneider - 2008^r10^ | pleural | 30 (30/70) | 95 (132/7) | 42 (42/58) | 95 (71/4) |  |  |
| 1.3 | SMRP | Creaney - 2008^r7^ | pleural | 73 (48/18) | 47 (14/16) | 73 (48/18) | 89 (62/8) |  |  |
| 1.4 | SMRP | Iwahori - 2008^r8^ | pleural | 59 (16/11) | 79 (65/17) | 59 (16/11) | 94 (44/3) |  |  |
| 1.5 | SMRP | Amati - 2008r6 ^ | pleural |  |  | 73 (16/6) | 90 (85/9) |  |  |
| 1.6 | SMRP | Pass - 2008^r9^ | pleural | 79 (71/19) | 76 (130/40) | 60 (54/36) | 89 (59/7) |  |  |
| 1.7 | SMRP | Van den Heuvel - 2007^r17^ | pleural | 60 (44/29) | 79 (84/22) |  |  |  |  |
| 1.8 | SMRP | Cristaudo - 2007^r13^ | pleural | 27 (29/78) | 95 (205/10) | 46 (49/58) | 94 (247/15) |  |  |
| 1.9 | SMRP | Di Serio - 2007^r14^ | pleural |  |  | 67 (16/8) | 92 (85/7) |  |  |
| 1.10 | SMRP | Scherpereel - 2005^r22^ | pleural | 58 (35/25) | 73 (22/8) | 80 (48/12) | 83 (19/4) |  |  |
| 2.1 | CEA* | Van den Heuvel - 2007^r17^ | pleural | 90 (66/7) | 52 (55/51) |  |  |  |  |
| 2.2 | CEA* | Fuhrman - 2000r27 ^ | pleural | 50 (13/13) | 88 (23/3) |  |  |  |  |
| 2.3 | CEA* | Alatas - 1999^r28^ | pleural | 55 (11/9) | 83 (20/4) |  |  |  |  |
| 2.4 | CEA* | Nisman - 1998^r30^ | pleural | 100 (14/0) | 48 (39/42) | 100 (14/0) | 0 (0/90) |  |  |
| 2.5 | CEA* | Ebert - 1997r32 ^ | pleural | 88 (29/4) | 39 (59/91) | 88 (29/4) | 2 (4/182) |  |  |
| 3.1 | CYFRA21-1 | Van den Heuvel - 2007^r17^ | pleural | 66 (48/25) | 33 (35/71) |  |  |  |  |
| 3.2 | CYFRA21-1 | Alatas - 1999^r28^ | pleural |  |  | 50 (10/10) | 74 (29/10) |  |  |
| 3.3 | CYFRA21-1 | Nisman - 1998^r30^ | pleural | 50 (7/7) | 56 (45/36) | 50 (7/7) | 93 (90/7) |  |  |
| 3.4 | CYFRA21-1 | Ebert - 1997r32 ^ | pleural | 36 (12/21) | 57 (85/65) | 36 (12/21) | 94 (182/12) |  |  |
| 4.1 | MPF | Creaney - 2008^r7^ | pleural | 32 (21/45) | 93 (28/2) | 32 (21/45) | 96 (67/3) |  |  |
| 4.2 | MPF | Iwahori - 2008^r8^ | pleural | 74 (20/7) | 84 (69/13) | 74 (20/7) | 98 (46/1) |  |  |
| 4.3 | MPF | Shiomi - 2007r16 ^ | pleural |  |  |  |  | 72 (28/11) | 93 (237/17) |
| 4.4 | MPF | Onda - 2006^r19^ | pleural, peritoneal |  |  | 91 (51/5) | 100 (70/0) |  |  |
| 5.1 | osteopontin | Creaney - 2008^r7^ | pleural | 45 (30/36) | 43 (13/17) | 45 (30/36) | 87 (61/9) |  |  |
| 5.2 | osteopontin | Grigoriu - 2007r15 ^ | pleural |  |  | 60 (56/38) | 80 (90/22) |  |  |
| 5.3 | osteopontin | Pass - 2005^r21^ | pleural |  |  | 78 (59/17) | 86 (59/10) |  |  |
| 6.1 | CA15-3 | Creaney - 2008^r81^ | pleural |  |  | 35 (17/32) | 85 (56/10) |  |  |
| 6.2 | CA15-3 | Alatas - 1999^r28^ | pleural |  |  | 80 (16/4) | 56 (20/16) |  |  |
| 7.1 | NSE* | Alatas - 1999^r28^ | pleural | 70 (14/6) | 63 (15/9) |  |  |  |  |

*sensitivity and specificity were calculated as follows: the number of mesothelioma patients below the cut-off value was defined as TP and those above the cut-off value as FN whereas the number of non-mesothelioma patients below the cut-off value was defined as FN and those above the cut-off value as TN; ^(r6) values of the asbestos exposed group were used to construct a two-by-two table because this was the largest comparison group; ^(r15) we estimated values from a ROC curve using a noticeable cut-off value that corresponded to a specificity of 80%; ^(r16) values from Figure 4A were extracted to construct the two-by-two table because it included the largest comparison group; ^(r27) for serum CEA, we estimated values from a ROC curve, using a noticeable cut-off value of 1 ng/ml; ^(r32) values were estimated from figure 1 to construct a two-by-two table.

*Table 2.1 continued*

| **No.** | **Marker** | **1st Author-year** | **Type of mesothelioma** | **in comparison with** | | | | | |
| --- | --- | --- | --- | --- | --- | --- | --- | --- | --- |
|  |  |  |  | **Malignancy** | | **Non-malignancy** | | **Malignancy and non-malignancy combined** | |
|  |  |  |  | **Sens %**  **TP/FN** | **Spec %**  **(TN/FP)** | **Sens %**  **TP/FN** | **Spec %**  **(TN/FP)** | **Sens %**  **TP/FN** | **Spec %**  **(TN/FP)** |
| 7.2 | NSE* | Ebert - 1997r32 ^ | pleural | 88 (29/4) | 17 (26/124) | 88 (29/4) | 2 (4/182) |  |  |
| 8.1 | p53 | Neri - 2003^r23^ | pleural | 7 (2/28) | 83 (40/8) | 7 (2/28) | 98 (104/2) |  |  |
| 8.2 | p53 | Creaney - 2001r25 ^ | unknown |  |  | 7 (6/82) | 94 (97/6) |  |  |
| 9.1 | TPS | Nisman - 1998^r30^ | pleural | 64 (9/5) | 65 (53/28) | 64 (9/5) | 91 (90/9) |  |  |
| 9.2 | TPS | Ebert - 1997r32 ^ | pleural | 36 (12/21) | 85 (127/23) | 36 (12/21) | 94 (182/12) |  |  |
| 10 | 80HdG | Amati - 2008r6 ^ | pleural |  |  | 18 (4/18) | 90 (85/9) |  |  |
| 11 | bFGF | Amati - 2008r6 ^ | pleural |  |  | 45 (10/12) | 90 (85/9) |  |  |
| 12 | CA125 | Creaney - 2007^r11^ | pleural |  |  | 42 (49/68) | 78 (91/25) |  |  |
| 13 | Gene-X | Shigematsu - 2009^r5^ | pleural | 56 (10/8) | 100 (63/0) | 56 (10/8) | 100 (25/0) |  |  |
| 14 | HA | Grigoriu - 2009^r3^ | pleural | 26 (20/56) | 76 (25/8) | 26 (20/56) | 96 (26/1) |  |  |
| 15 | HGF | Amati - 2008r6 ^ | pleural |  |  | 36 (8/14) | 90 (85/9) |  |  |
| 16 | PDGF-AB | Filiberti - 2005^r20^ | pleural | 43 (40/53) | 70 (23/10) | 43 (40/53) | 82 (42/9) |  |  |
| 17 | PDGFß | Amati - 2008r6 ^ | pleural |  |  | 45 (10/12) | 90 (85/9) |  |  |
| 18 | THBS-2 | Shigematsu - 2009^r5^ | pleural | 89 (16/2) | 100 (63/0) | 89 (16/2) | 92 (23/2) |  |  |
| 19 | TPA-M* | Ebert - 1997r32 ^ | pleural | 76 (25/8) | 39 (59/91) | 76 (25/8) | 4 (8/182) |  |  |
| 20 | TSA | Alatas - 1999^r28^ | pleural | 40 (8/12) | 25 (6/18) |  |  |  |  |
| 21 | VEGFß | Amati - 2008r6 ^ | pleural |  |  | 59 (13/9) | 90 (85/9) |  |  |

*sensitivity and specificity were calculated as follows: the number of mesothelioma patients below the cut-off value was defined as TP and those above the cut-off value as FN whereas the number of non-mesothelioma patients below the cut-off value was defined as FN and those above the cut-off value as TN; ^(r6) values of the asbestos exposed group were used to construct a two-by-two table because this was the largest comparison group; ^(r25) values were additionally extracted from figure 2 to construct a two-by-two table for the comparison of mesothelima to non-malignancy; ^(r32) values were estimated from figure 1 to construct a two-by-two table.

Table 2.2: Reported sensitivity and specificity of effusion markers per study, stratified by type marker

| **No.** | **Marker** | **1st Author-year** | **Type of effusion(s)** | **in comparison with** | | | | | |
| --- | --- | --- | --- | --- | --- | --- | --- | --- | --- |
|  |  |  |  | **Malignancy** | | **Non-malignancy** | | **Malignancy and non-malignancy combined** | |
|  |  |  |  | **Sens %**  **TP/FN** | **Spec %**  **(TN/FP)** | **Sens %**  **TP/FN** | **Spec %**  **(TN/FP)** | **Sens %**  **TP/FN** | **Spec %**  **(TN/FP)** |
| 1.1 | CEA* | Villena - 2003^r24^ | pleural | 100 (20/0) | 43 (35/46) | 100 (20/0) | 0 (0/151) |  |  |
| 1.2 | CEA* | Paganuzzi - 2001^r26^ | pleural | 97 (31/1) | 53 (21/19) | 97 (31/1) | 9 (3/31) |  |  |
| 1.3 | CEA* | Fuhrman - 2000^r27^ | pleural | 100 (29/0) | 76 (25/8) |  |  |  |  |
| 1.4 | CEA* | Alatas - 1999^r28^ | pleural | 90 (18/2) | 88 (21/3) |  |  |  |  |
| 1.5 | CEA* | Miedouge - 1999^r29^ | pleural | 100 (11/0) | 63 (129/75) | 100 (11/0) | 1 (1/120) |  |  |
| 1.6 | CEA* | Atagi - 1997^r31^ | pleural | 77 (10/3) | 68 (17/8) |  |  |  |  |
| 1.7 | CEA* | Villena - 1995^r34^ | pleural | 100 (10/0) | 51 (28/27) | 100 (10/0) | 0 (0/142) |  |  |
| 1.8 | CEA* | Shijubo - 1995^r33^ | pleural | 100 (10/0) | 68 (53/25) |  |  |  |  |
| 1.9 | CEA* | Whitaker - 1986^r35^ | pleural | 100 (20/0) | 70 (14/6) | 100 (20/0) | 0 (0/20) |  |  |
| 1.10 | CEA* | Fravelli - 1984^r36^ | pleural | 100 (26/0) | 68 (77/37) | 100 (26/0) | 33 (24/49) |  |  |
| 2.1 | CA15-3 | Creaney - 2008^r81^ | pleural | 38 (20/32) | 76 (19/6) | 38 (20/32) | 100 (30/0) |  |  |
| 2.2 | CA15-3 | Villena - 2003^r24^ | pleural | 30 (6/14) | 56 (45/36) | 30 (6/14) | 100 (151/0) |  |  |
| 2.3 | CA15-3 | Miedouge - 1999^r29^ | pleural | 45 (5/6) | 35 (72/132) | 45 (5/6) | 99 (120/1) |  |  |
| 2.4 | CA15-3 | Alatas - 1999^r28^ | pleural |  |  | 90 (18/2) | 93 (28/2) |  |  |
| 2.5 | CA15-3 | Villena - 1995^r34^ | pleural | 70 (7/3) | 47 (26/29) | 70 (7/3) | 100 (142/0) |  |  |
| 3.1 | HA | Grigoriu - 2009^r3^ | pleural |  |  |  |  | 64 (49/27) | 97 (58/2) |
| 3.2 | HA | Welker - 2007r18 ^ | pleural | 88 (63/9) | 99 (99/1) | 88 (63/9) | 97 (87/3) |  |  |
| 3.3 | HA | Dejmek - 2005^r82^ | unknown | 35 (20/37) | 100 (73/0) | 35 (20/37) | 100 (36/0) |  |  |
| 3.4 | HA | Fuhrman - 2000^r27^ | pleural | 32 (12/25) | 95 (36/2) |  |  |  |  |
| 3.5 | HA | Atagi - 1997^r31^ | pleural |  |  |  |  | 37 (7/12) | 99 (79/1) |
| 4.1 | SMRP | Davies - 2009^r2^ | pleural | 71 (17/7) | 81 (54/13) | 71 (17/7) | 97 (73/2) |  |  |
| 4.2 | SMRP | Aleman - 2009^r1^ | pleural |  |  |  |  | 56 (10/8) | 92 (46/4) |
| 4.3 | SMRP | Pass - 2008^r9^ | pleural | 76 (34/11) | 44 (16/20) | 76 (34/11) | 83 (25/5) |  |  |
| 4.4 | SMRP | Creaney - 2007r12 ^ | pleural, peritoneal | 68 (40/19) | 83 (58/12) | 68 (40/19) | 98 (88/2) |  |  |
| 4.5 | SMRP | Scherpereel - 2005^r22^ | pleural | 58 (25/18) | 93 (26/2) | 77 (33/10) | 24 (5/16) |  |  |
| 5.1 | CA19-9* | Alatas - 1999^r28^ | pleural | 70 (14/6) | 54 (13/11) |  |  |  |  |
| 5.2 | CA19-9* | Miedouge - 1999^r29^ | pleural | 100 (11/0) | 22 (45/159) | 100 (11/0) | 1 (1/120) |  |  |
| 5.3 | CA19-9* | Villena - 1995^r34^ | pleural | 100 (10/0) | 24 (13/42) | 100 (10/0) | 0 (0/142) |  |  |
| 6.1 | CA72-4* | Villena - 2003^r24^ | pleural | 100 (20/0) | 37 (30/51) | 100 (20/0) | 0 (0/151) |  |  |
| 6.2 | CA72-4* | Miedouge - 1999^r29^ | pleural | 100 (11/0) | 72 (147/57) | 100 (11/0) | 1 (1/120) |  |  |
| 6.3 | CA72-4* | Villena - 1995^r34^ | pleural | 90 (9/1) | 58 (32/23) | 90 (9/1) | 2 (3/139) |  |  |

*sensitivity and specificity were calculated as follows: the number of mesothelioma patients below the cut-off value was defined as TP and those above the cut-off value as FN whereas the number of non-mesothelioma patients below the cut-off value was defined as FN and those above the cut-off value as TN; ^(r12) values of pleural and peritoneal effusions were combined; ^(r18) combining the results of table 1 and figure 2 and using a cut-off value of 100 mg/l, we could construct a separate two-by-two table for the comparison of mesothelioma to non-malignancy and malignancy.

*Table 2.2 continued*

| **No.** | **Marker** | **1st Author-year** | **Type of effusion(s)** | **in comparison with** | | | | | |
| --- | --- | --- | --- | --- | --- | --- | --- | --- | --- |
|  |  |  |  | **Malignancy** | | **Non-malignancy** | | **Malignancy and non-malignancy combined** | |
|  |  |  |  | **Sens %**  **TP/FN** | **Spec %**  **(TN/FP)** | **Sens %**  **TP/FN** | **Spec %**  **(TN/FP)** | **Sens %**  **TP/FN** | **Spec %**  **(TN/FP)** |
| 7.1 | CYFRA21-1 | Paganuzzi - 2001^r26^ | pleural | 88 (28/4) | 33 (13/27) | 88 (28/4) | 79 (27/7) |  |  |
| 7.2 | CYFRA21-1 | Alatas - 1999^r28^ | pleural |  |  | 90 (18/2) | 90 (27/3) |  |  |
| 7.3 | CYFRA21-1 | Miedouge - 1999^r29^ | pleural | 55 (6/5) | 55 (112/92) | 55 (6/5) | 99 (120/1) |  |  |
| 8.1 | NSE* | Alatas - 1999^r28^ | pleural | 80 (16/4) | 63 (15/9) |  |  |  |  |
| 8.2 | NSE* | Miedouge - 1999^r29^ | pleural | 91 (10/1) | 19 (38/166) | 91 (10/1) | 2 (3/118) |  |  |
| 9 | CA549* | Villena - 2003^r24^ | pleural | 65 (13/7) | 46 (37/44) | 65 (13/7) | 0 (0/151) |  |  |
| 10 | SCC* | Miedouge - 1999^r29^ | pleural | 100 (11/0) | 6 (12/192) | 100 (11/0) | 1 (1/120) |  |  |
| 11 | SP-A* | Shijubo - 1995^r33^ | pleural | 100 (10/0) | 47 (37/41) |  |  |  |  |
| 12 | TSA | Alatas - 1999^r28^ | pleural | 90 (18/2) | 50 (12/12) |  |  |  |  |

*sensitivity and specificity were calculated as follows: the number of mesothelioma patients below the cut-off value was defined as TP and those above the cut-off value as FN whereas the number of non-mesothelioma patients below the cut-off value was defined as FN and those above the cut-off value as TN.

Table 2.3 Reported sensitivity and specificity of immunohistochemical markers per study stratified by type marker

| **No.** | **Marker** | **1st Author-year** | **Type of effusion(s)** | **In comparison with** | | | |
| --- | --- | --- | --- | --- | --- | --- | --- |
|  |  |  |  | **Malignancy** | | **Non-malignancy** | |
|  |  |  |  | **Sens %**  **TP/FN** | **Spec %**  **(TN/FP)** | **Sens %**  **TP/FN** | **Spec %**  **(TN/FP)** |
| 1.1 | EMA | Shen - 2009^r37^ | pleural, peritoneal |  |  | 86 (30/5) | 87 (33/5) |
| 1.2 | EMA (any staining) | Yuan - 2009r39 ^ | pleural, peritoneal, pericardial | 97 (35/1) | 0 (0/94) |  |  |
| 1.2 | EMA (membranous staining) | Yuan - 2009^r39^ | pleural, peritoneal, pericardial | 92 (33/3) | 100 (94/0) |  |  |
| 1.3 | EMA (E29) | Creaney - 2008r81 ^ | pleural |  |  | 84 (16/3) | 93 (14/1) |
| 1.3 | EMA (Mc5) | Creaney - 2008r81 ^ | pleural |  |  | 100 (20/0) | 0 (0/14) |
| 1.4 | EMA (any staining) | Grefte - 2007^r42^ | pleural, peritoneal | 100 (11/0) | 0 (0/12) | 100 (11/0) | 91 (10/1) |
| 1.4 | EMA (membranous staining) | Grefte - 2007r42 ^ | pleural, peritoneal | 91 (10/1) | 100 (12/0) | 91 (10/1) | 91 (10/1) |
| 1.5 | EMA | Aerts - 2006^r46^ | pleural | 86 (12/2) | 0 (0/12) | 86 (12/2) | 100 (13/0) |
| 1.6 | EMA (E29) | Saad - 2005^r53^ | pleural |  |  | 75 (15/5) | 100 (20/0) |
| 1.6 | EMA (Mc5) | Saad - 2005^r53^ | pleural |  |  | 70 (14/6) | 60 (12/8) |
| 1.7 | EMA (any staining) | Dejmek - 2005^r82^ | unknown | 73 (40/15) | 8 (11/121) |  |  |
| 1.7 | EMA (membranous staining) | Dejmek - 2005^r82^ | unknown | 58 (32/23) | 99 (123/1) |  |  |
| 1.8 | EMA | Motherby - 1999^r64^ | pleural, peritoneal, pericardial, cul de sac | 100 (14/0) | 2 ^#^ (2/85) | 100 (14/0) | 71 ^$^ (37/15) |
| 1.9 | EMA | Ascoli - 1995r68 ^ | pleural, peritoneal, hydrocoele, synovial | 100 (33/0) | 9 (13/139) | 100 (33/0) | 91 ^$^ (32/3) |
| 1.10 | EMA | Delahaye - 1991^r72^ | pleural, peritoneal | 83 (20/4) | 0 (0/31) | 83 (20/4) | 100 (20/0) |
| 1.11 | EMA (any staining) | Cibas - 1987^r75^ | pleural, peritoneal | 95 (19/1) | 5 (2/37) |  |  |
| 1.11 | EMA (membranous staining) | Cibas - 1987r75 ^ | pleural, peritoneal | 55 (11/9) | 82 (32/7) |  |  |
| 2.1 | Ber-EP4* | Yuan - 2009^r39^ | pleural, peritoneal, pericardial | 64 (23/13) | 98 (92/2) |  |  |
| 2.2 | Ber-EP4* | Botelho - 2008^r80^ | pleural, peritoneal | 84 (27/5) | 87 (27/4) | 84 (27/5) | 0 (0/39) |
| 2.3 | Ber-EP4* | Grefte - 2007^r42^ | pleural, peritoneal | 100 (11/0) | 92 (11/1) | 100 (11/0) | 0 (0/11) |
| 2.4 | Ber-EP4* | Aerts - 2006^r46^ | pleural | 93 (13/1) | 100 (12/0) | 93 (13/1) | 0 (0/13) |
| 2.5 | Ber-EP4* | Li - 2006^r48^ | pleural | 100 (12/0) | 48 (10/11) |  |  |
| 2.6 | Ber-EP4* | Dejmek - 2005^r82^ | unknown | 84 (46/9) | 56 (77/61) |  |  |
| 2.7 | Ber-EP4* | Motherby - 1999^r64^ | pleural, peritoneal, pericardial, cul de sac | 100 (14/0) | 95 ^#^ (83/4) | 100 (14/0) | 0 ^$^ (0/53) |
| 2.8 | Ber-EP4* | Delahaye - 1997^r67^ | unknown | 98 (40/1) | 78 (69/19) | 98 (40/1) | 0 (0/25) |
| 2.9 | Ber-EP4* | Ascoli - 1995r68 ^ | pleural, peritoneal, hydrocoele, synovial | 100 (33/0) | 95 (145/7) | 100 (33/0) | 0 ^$^ (0/35) |
| 2.10 | Ber-EP4* | Kuhlman - 1991^r73^ | pleural | 85 (17/3) | 75 (15/5) | 100 (20/0) | 0 ^$^ (0/20) |
| 3.1 | CEA* | Grefte - 2007^r42^ | pleural, peritoneal | 100 (11/0) | 58 (7/5) | 100 (11/0) | 0 (0/11) |
| 3.2 | CEA* | Aerts - 2006^r46^ | pleural | 93 (13/1) | 92 (11/1) | 93 (13/1) | 0 (0/13) |

*sensitivity and specificity were calculated as follows: the number of mesothelioma patients below the cut-off value was defined as TP and those above the cut-off value as FN whereas the number of non-mesothelioma patients below the cut-off value was defined as FN and those above the cut-off value as TN; ^(r39) for any staining pattern, a two-by-two table was constructed by combining the values of membranous and cytoplasmic staining; ^(r42, r75) membranous staining was classified as a predominant staining of the mebrame (i.e. the membranous staining was substantially greater than the cytoplasmic); ^#^(r64) values of other malignant diseases included two patients of chronic unspecific pleuritis; ^$^(r64) values of non-malignancy included one patient with lung cancer; ^(r68) the values of group IV were excluded from the two-by-two table because these were highly influenced by incorporation bias; ^$ (^r68) 40% of the samples that contained benign reactive cells were obtained from patients with underlying malignancies; ^$^(r73) we considered samples with benign reactive cells as benign, although the underlying diseases were not described; ^(r81) other anti-EMA clones were also studied, but E29 and Mc5 were the most useful clones.

Table 2.3 continued

| **No.** | **Marker** | **1st Author-year** | **Type of effusion(s)** | **In comparison with** | | | |
| --- | --- | --- | --- | --- | --- | --- | --- |
|  |  |  |  | **Malignancy** | | **Non-malignancy** | |
|  |  |  |  | **Sens %**  **TP/FN** | **Spec %**  **(TN/FP)** | **Sens %**  **TP/FN** | **Spec %**  **(TN/FP)** |
| 3.3 | CEA* | Li - 2006^r48^ | pleural | 100 (12/0) | 57 (12/9) |  |  |
| 3.4 | CEA* | Dejmek - 2005^r82^ | unknown | 98 (49/1) | 68 (93/44) |  |  |
| 3.5 | CEA* | Davidson - 2001^r58^ | pleural, peritoneal | 100 (12/0) | 41 (40/58) | 100 (12/0) | 0 (0/56) |
| 3.6 | CEA* | Motherby - 1999^r64^ | pleural, peritoneal, pericardial, cul de sac | 100 (14/0) | 49 ^#^ (42/43) | 100 (14/0) | 8 ^$^ (4/48) |
| 3.7 | CEA* | Delahaye - 1997^r67^ | unknown | 100 (41/0) | 55 (48/40) | 100 (41/0) | 0 (0/25) |
| 3.8 | CEA* | Kuhlman - 1991^r73^ | pleural | 90 (18/2) | 50 (10/10) | 100 (20/0) | 0 ^$^ (0/20) |
| 3.9 | CEA* | Ghosh - 1987^r76^ | pleural, peritoneal | 92 (11/1) | 75 (6/2) | 92 (11/1) | 0  (0/5) |
| 3.10 | CEA* | Cibas - 1987^r75^ | pleural, peritoneal | 100 (20/0) | 72 (28/11) |  |  |
| 4.1 | calretinin | Yuan - 2009^r39^ | pleural, peritoneal, pericardial | 97 (35/1) | 79 (74/20) |  |  |
| 4.2 | calretinin | Botelho - 2008^r80^ | pleural, peritoneal | 100 (32/0) | 100 (31/0) | 100 (32/0) | 5 (2/37) |
| 4.3 | calretinin | Grefte - 2007^r42^ | pleural, peritoneal | 91 (10/1) | 100 (12/0) | 91 (10/1) | 0 (0/11) |
| 4.4 | calretinin | Shield - 2007^r45^ | pleural, peritoneal | 97 (33/1) | 97 (65/2) |  |  |
| 4.5 | calretinin | Bhalla - 2007^r40^ | pleural, peritoneal | 100 (10/0) | 100 (10/0) | 100 (10/0) | 0 (0/20) |
| 4.6 | calretinin | Saad - 2006^r49^ | pleural | 85 (17/3) | 55 (11/9) |  |  |
| 4.7 | calretinin | Li - 2006^r48^ | pleural | 100 (12/0) | 100 (21/0) |  |  |
| 4.8 | calretinin | Wieczorek - 2000r62 ^ | pleural, peritoneal | 100 (29/0) | 62 (24/15) |  |  |
| 4.9 | calretinin | Simir - 1999^r65^ | pleural | 58 (15/11) | 69 (20/9) | 58 (15/11) | 73 ^$^ (16/6) |
| 5.1 | B72-3* | Yuan - 2009^r39^ | pleural, peritoneal, pericardial | 100 (36/0) | 69 (65/29) |  |  |
| 5.2 | B72-3* | Grefte - 2007^r42^ | pleural, peritoneal | 100 (11/0) | 42 (5/7) | 100 (11/0) | 0 (0/11) |
| 5.3 | B72-3* | Delahaye - 1997^r67^ | unknown | 98 (40/1) | 77 (68/20) | 98 (40/1) | 0 (0/25) |
| 5.4 | B72-3* | Kuhlman - 1991^r73^ | pleural | 90 (18/2) | 95 (19/1) | 100 (20/0) | 0 ^$^ (0/20) |
| 5.5 | B72-3* | Betta - 1991^r71^ | pleural, peritoneal | 90 (9/1) | 80 (16/4) |  |  |
| 6.1 | HMFG-2* | Grefte - 2007^r42^ | pleural, peritoneal | 36 (4/7) | 100 (12/0) | 36 (4/7) | 0 (0/11) |
| 6.2 | HMFG-2* | Linari - 1989r74 ^ | pleural, peritoneal, pericardial | 9 (1/10) | 71 (146/60) | 9 (1/10) | 0 (0/15) |
| 6.3 | HMFG-2* | Ghosh - 1987^r76^ | pleural, peritoneal | 17 (2/10) | 63 (5/3) | 17 (2/10) | 0  (0/5) |
| 6.4 | HMFG-2* | Cibas - 1987^r75^ | pleural, peritoneal | 20 (4/16) | 95 (37/2) |  |  |
| 7.1 | cytokeratin CK5/6 | Shield - 2007^r45^ | pleural, peritoneal | 97 (33/1) | 91 (61/6) |  |  |
| 7.2 | cytokeratin CK5/6 | Saad - 2006^r49^ | pleural | 90 (18/2) | 50 (10/10) |  |  |
| 7.3 | cytokeratin CK5/6 | Li - 2006^r48^ | pleural | 92 (11/1) | 57 (12/9) |  |  |
| 8.1 | D2-40 | Bhalla - 2007^r40^ | pleural, peritoneal | 100 (10/0) | 100 (10/0) | 100 (10/0) | 20 (4/16) |

*sensitivity and specificity were calculated as follows: the number of mesothelioma patients below the cut-off value was defined as TP and those above the cut-off value as FN whereas the number of non-mesothelioma patients below the cut-off value was defined as FN and those above the cut-off value as TN; ^(r62) samples of papillary serous borderline malignancies were excluded from the two-by-two table because these were taken from tissue or washings; ^#^(r64) values of other malignant diseases included two patients of chronic unspecific pleuritis; ^$^(r64) values of non-malignancy included one patient with lung cancer; ^$^(r65) we considered samples with benign reactive cells as benign, although the underlying diseases were not described; ^$^(r73) we considered samples with benign reactive cells as benign, although the underlying diseases were not described; ^(r74) patients with an inconclusive HMFG-2 test or a doubtful diagnosis were not considered in the two-by-two table.

*Table 2.3 continued*

| **No.** | **Marker** | **1st Author-year** | **Type of effusion(s)** | **In comparison with** | | | |
| --- | --- | --- | --- | --- | --- | --- | --- |
|  |  |  |  | **Malignancy** | | **Non-malignancy** | |
|  |  |  |  | **Sens %**  **TP/FN** | **Spec %**  **(TN/FP)** | **Sens %**  **TP/FN** | **Spec %**  **(TN/FP)** |
| 8.2 | D2-40 | Saad - 2006^r49^ | pleural | 85 (17/3) | 100 (20/0) |  |  |
| 8.3 | D2-40 | Bassarova - 2006^r47^ | pleural, peritoneal, pericardial | 94 (30/2) | 92 (230/20) | 94 (30/2) | 0  (0/8) |
| 9.1 | Leu-M1* | Dejmek - 1999^r63^ | pleural | 86 (30/5) | 51 (24/23) | 86 (30/5) | 4 (1/23) |
| 9.2 | Leu-M1* | Motherby - 1999^r64^ | pleural, peritoneal, pericardial, cul de sac | 100 (14/0) | 32 ^#^ (28/59) | 100 (14/0) | 0 ^$^ (0/53) |
| 9.3 | Leu-M1* | Delahaye - 1997^r67^ | unknown | 100 (41/0) | 28 (25/63) | 100 (41/0) | 0 (0/25) |
| 10.1 | MOC-31* | Pu - 2007^r44^ | pleural, peritoneal | 67 (12/6) | 80 (20/5) |  |  |
| 10.2 | MOC-31* | Hecht - 2005^r52^ | pleural, peritoneal, pericardial | 86 (6/1) | 100 (86/0) | 94 (16/1) | 11 (1/8) |
| 10.3 | MOC-31* | Delahaye - 1997^r67^ | unknown | 88 (36/5) | 76 (67/21) | 88 (36/5) | 0 (0/25) |
| 11.1 | TTF-1* | Saad - 2006^r49^ | pleural | 100 (20/0) | 45 (9/11) |  |  |
| 11.2 | TTF-1* | Afify - 2002^r56^ | unknown | 100 (12/0) | 39 (27/43) |  |  |
| 11.3 | TTF-1* | Hecht - 2001r60 ^ | pleural, peritoneal, pericardial | 100 (14/0) | 39 (37/57) |  |  |
| 12.1 | vimentin | Dejmek - 2005^r82^ | unknown | 84 (43/8) | 50 (60/59) |  |  |
| 12.2 | vimentin | Davidson - 2001^r58^ | pleural, peritoneal | 75 (9/3) | 67 (66/32) | 75 (9/3) | 11 (6/50) |
| 12.3 | vimentin | Kuhlman - 1991^r73^ | pleural | 95 (19/1) | 80 (16/4) | 85 (17/3) | 15 ^$^ (3/17) |
| 13.1 | Ca125 | Dejmek - 2005^r82^ | unknown | 92 (12/1) | 34 (24/46) |  |  |
| 13.2 | CA125 | Davidson - 2001^r58^ | pleural, peritoneal | 100 (12/0) | 29 (28/70) | 100 (12/0) | 16 (9/47) |
| 14.1 | cytokeratin | Ascoli - 1995r68 ^ | pleural, peritoneal, hydrocoele, synovial | 100 (33/0) | 5 (7/145) | 100 (33/0) | 0 ^$^  (0/35) |
| 14.2 | cytokeratin | Kuhlman - 1991^r73^ | pleural | 100 (20/0) | 0 (0/20) | 95 (19/1) | 40 ^$^ (8/12) |
| 15.1 | cytokeratin CAM5-2 | Li - 2006^r48^ | pleural | 100 (12/0) | 10 (2/19) |  |  |
| 15.2 | cytokeratin CAM5-2 | Dejmek - 1999^r63^ | pleural | 97 (33/1) | 2 (1/50) | 97 (33/1) | 0 (0/24) |
| 16.1 | desmin* | Afify - 2002^r57^ | pleural, peritoneal | 100 (14/0) | 0 (0/56) | 100 (14/0) | 92 ^$^ (22/2) |
| 16.2 | desmin* | Davidson - 2001^r58^ | pleural, peritoneal | 92 (11/1) | 2 (2/96) | 92 (11/1) | 84 (47/9) |
| 17.1 | E-cadherin | Sivertsen - 2006^r50^ | pleural, peritoneal | 58 (14/10) | 13 (7/46) |  |  |
| 17.2 | E-cadherin | Simir - 1999^r65^ | pleural | 46 (12/14) | 3 (1/28) | 46 (12/14) | 86 ^$^ (19/3) |
| 18.1 | HBME-1 | Dejmek - 2005^r82^ | unknown | 0  (0/7) | 23 (14/47) |  |  |
| 18.2 | HBME-1 | Ascoli - 1997r66 ^ | pleural, peritoneal, pericardial, hydrocoele | 100 (47/0) | 76 (95/30) |  |  |
| 19.1 | keratin | Cibas - 1987^r75^ | pleural, peritoneal | 100 (20/0) | 0 (0/39) |  |  |
| 19.2 | keratin (kDalton63) | Walts - 1983r77 ^ | unknown |  |  | 83 (10/2) | 0 (0/15) |
| 20.1 | mesothelin | Pu - 2007^r44^ | pleural, peritoneal | 44 (8/10) | 24 (6/19) |  |  |

*sensitivity and specificity were calculated as follows: the number of mesothelioma patients below the cut-off value was defined as TP and those above the cut-off value as FN whereas the number of non-mesothelioma patients below the cut-off value was defined as FN and those above the cut-off value as TN; ^$^(r57, r73, r65) we considered samples with benign reactive cells as benign, although the underlying diseases were not described; ^(r60) FNA samples (i.e. all primary lung cancers) were excluded from the two-by-two table;  ^#^(r64) values of other malignant diseases included two patients of chronic unspecific pleuritis;  ^$^(r64) values of non-malignancy included one patient with lung cancer; ^(r66) controversial cases were excluded from the two-by-two table because the final diagnosis in these patients was uncertain; ^(r68) the values of group IV were excluded from the two-by- two table because these were highly influenced by incorporation bias; ^$(^r68) 40% of the samples that contained benign reactive cells were obtained from patients with underlying malignancies; ^(r77) a two-by-two table was not constructed for the comparison of mesothelioma to adenocarcinomas because >10% of the carcinoma samples were obtained by FNAs. kDalton45 showed similar values;

*Table 2.3 continued*

| **No.** | **Marker** | **1st Author-year** | **Type of effusion(s)** | **In comparison with** | | | |
| --- | --- | --- | --- | --- | --- | --- | --- |
|  |  |  |  | **Malignancy** | | **Non-malignancy** | |
|  |  |  |  | **Sens %**  **TP/FN** | **Spec %**  **(TN/FP)** | **Sens %**  **TP/FN** | **Spec %**  **(TN/FP)** |
| 20.2 | mesothelin | Donna - 1992^r70^ | pleural, peritoneal | 100 (12/0) | 100 (12/0) |  |  |
| 21.1 | N-cadherin | Sivertsen - 2006^r50^ | pleural, peritoneal | 63 (15/9) | 25 (13/40) |  |  |
| 21.2 | N-cadherin | Simir - 1999^r65^ | pleural | 35 (9/17) | 52 (15/14) | 35 (9/17) | 23 ^$^ (5/17) |
| 22.1 | p63* | Pu - 2007^r44^ | pleural, peritoneal | 100 (18/0) | 60 (15/10) |  |  |
| 22.2 | p63* | Saad - 2006^r49^ | pleural | 100 (20/0) | 55 (11/9) |  |  |
| 23.1 | thrombomodulin | Dejmek - 2005^r82^ | unknown | 86 (6/1) | 53 (31/28) |  |  |
| 23.2 | thrombomodulin | Ascoli - 1995r68 ^ | pleural, peritoneal, hydrocoele, synovial | 100 (33/0) | 62 (94/58) | 100 (33/0) | 0 ^$^  (0/35) |
| 24.1 | WT-1 | Pu - 2007^r44^ | pleural, peritoneal | 100 (18/0) | 100 (25/0) |  |  |
| 24.2 | WT-1 | Saad - 2006^r49^ | pleural | 95 (19/1) | 100 (20/0) |  |  |
| 25 | actin* | Afify - 2002^r57^ | pleural, peritoneal | 100 (14/0) | 0 (0/56) | 100 (14/0) | 0 ^$^ (0/24) |
| 26 | BMA-120 | Kuhlman - 1991^r73^ | pleural | 85 (17/3) | 90 (18/2) | 80 (16/4) | 20 ^$^ (4/16) |
| 27 | CA1/2 | Ghosh - 1987^r76^ | pleural, peritoneal | 75 (9/3) | 38 (3/5) | 75 (9/3) | 100 (5/0) |
| 28 | CD138* | Saqi - 2005r54 ^ | pleural, peritoneal, pericardial | 92 (22/2) | 44 (19/24) | 92 (22/2) | 0 (0/8) |
| 29 | CD44S | Afify - 2005^r51^ | pleural, peritoneal | 86 (12/2) | 62 (38/23) | 86 (12/2) | 0 ^$^ (0/28) |
| 30 | claudin1* | Kleinberg - 2007^r43^ | pleural, peritoneal, pericardial | 80 (20/5) | 71 (212/88) |  |  |
| 31 | claudin3* | Kleinberg - 2007^r43^ | pleural, peritoneal, pericardial | 100 (25/0) | 59 (177/123) |  |  |
| 32 | claudin4* | Facchetti - 2007^r41^ | pleural, peritoneal | 100 (23/0) | 97 (60/2) | 100 (23/0) | 0 ^$^ (0/12) |
| 33 | cytokeratin CK5 | Bhalla - 2007^r40^ | pleural, peritoneal | 100 (10/0) | 90 (9/1) | 100 (10/0) | 0 (0/20) |
| 34 | cytokeratin K903 | Li - 2006^r48^ | pleural | 92 (11/1) | 52 (11/10) |  |  |
| 35 | Glut-1m | Shen - 2009^r37^ | pleural, peritoneal |  |  | 63 (22/13) | 82 (31/7) |
| 36 | Glut-1p | Shen - 2009^r37^ | pleural, peritoneal |  |  | 83 (29/6) | 63 (24/14) |
| 37 | HA | Afify - 2005^r51^ | pleural, peritoneal | 100 (14/0) | 100 (61/0) | 100 (14/0) | 7 ^$^ (2/26) |
| 38 | HEA-125* | Kuhlman - 1991^r73^ | pleural | 95 (19/1) | 95 (19/1) | 100 (20/0) | 0 ^$^ (0/20) |
| 39 | IGF-II* | Slipicevic - 2009^r38^ | pleural, peritoneal, pericardial | 70 (23/10) | 59 (172/122) |  |  |
| 40 | IGFBP3* | Slipicevic - 2009^r38^ | pleural, peritoneal, pericardial | 76 (25/8) | 49 (144/150) |  |  |
| 41 | keratin7* | Baars - 1994^r69^ | pleural, peritoneal | 100 (10/0) | 73 (46/17) | 100 (10/0) | 20 ^$^ (3/12) |
| 42 | Ki67 | Schonherr - 2004^r55^ | pleural, peritoneal, pericardial |  |  | 25 (5/15) | 100 ^$^ (20/0) |
| 43 | myogenin* | Afify - 2002^r57^ | pleural, peritoneal | 100 (14/0) | 0 (0/56) | 100 (14/0) | 0 ^$^ (0/24) |
| 44 | myoglobin* | Afify - 2002^r57^ | pleural, peritoneal | 100 (14/0) | 0 (0/56) | 100 (14/0) | 0 ^$^ (0/24) |
| 45 | OV632 | Delahaye - 1991^r72^ | pleural, peritoneal | 92 (22/2) | 68 (21/10) | 92 (22/2) | 100 (20/0) |

*sensitivity and specificity were calculated as follows: the number of mesothelioma patients below the cut-off value was defined as TP and those above the cut-off value as FN whereas the number of non-mesothelioma patients below the cut-off value was defined as FN and those above the cut-off value as TN; ^$^(r41, r51, r57, r65, r69, r73) we considered samples with benign reactive cells as benign, although the underlying diseases were not described; ^(r54) pelvic washings were excluded from the two-by-two table. The ovary and uturus malignancies were completely excluded because seperate values for pelvic washings and effusions could not be obtained; ^$^(r55) underlying diseases of cytologic benign effusions included also patients with malignancies; ^(r68) the values of group IV were excluded from the two-by-two table because these were highly influenced by incorporation bias; ^$(^r68) 40% of the samples that contained benign reactive cells were obtained from patients with underlying malignancies.

*Table 2.3 continued*

| **No.** | **Marker** | **1st Author-year** | **Type of effusion(s)** | **In comparison with** | | | |
| --- | --- | --- | --- | --- | --- | --- | --- |
|  |  |  |  | **Malignancy** | | **Non-malignancy** | |
|  |  |  |  | **Sens %**  **TP/FN** | **Spec %**  **(TN/FP)** | **Sens %**  **TP/FN** | **Spec %**  **(TN/FP)** |
| 46 | P-cadherin | Sivertsen - 2006^r50^ | pleural, peritoneal | 83 (20/4) | 38 (20/33) |  |  |
| 47 | p53 | Davidson - 2001^r58^ | pleural, peritoneal | 83 (10/2) | 33 (32/66) | 83 (10/2) | 86 (48/8) |
| 48 | podoplanin | Bhalla - 2007^r40^ | pleural, peritoneal | 100 (10/0) | 100 (10/0) | 100 (10/0) | 15 (3/17) |
| 49 | Sial-Tn | Dejmek - 2005^r82^ | unknown | 71 (5/2) | 52 (30/28) |  |  |
| 50 | SV-40* | Simsir - 2001^r61^ | pleural | 100 (32/0) | 0 (0/43) | 100  32/0) | 0 (0/25) |
| 51 | TAG-72* | Aerts - 2006^r46^ | pleural | 100 (14/0) | 92 (11/1) | 100 (14/0) | 0 (0/13) |
| 52 | Tenascin-X | Yuan - 2009^r39^ | pleural, peritoneal, pericardial | 76 (28/9) | 97 (133/4) | 76 (28/9) | 89 ^$^ (8/1) |
| 53 | WT1 | Hecht - 2001^r59^ | pleural, peritoneal, pericardial | 100 (14/0) | 77 (75/22) |  |  |
| 54 | XIAP | Shen - 2009^r37^ | pleural, peritoneal |  |  | 83 (29/6) | 39 (15/23) |

^$^(r39) we considered samples with benign reactive cells as benign, although the underlying diseases were not described.

Table 2.4 Reported sensitivity and specificity of genetic markers in effusion per study stratified by type

| **No.** | **Marker** | **1st Author-year** | **Type of effusion^(s)^** | **In comparison with** | | | |
| --- | --- | --- | --- | --- | --- | --- | --- |
|  |  |  |  | **Malignancy** | | **Non-malignancy** | |
|  |  |  |  | **Sens %**  **TP/FN** | **Spec %**  **(TN/FP)** | **Sens %**  **TP/FN** | **Spec %**  **(TN/FP)** |
| 1.1 | CDKN2A-deletion | Flores-Staino - 2009^r79^ | pleural | 57 (12/9) | 90 (26/3) | 57 (12/9) | 100 (18/0) |
| 1.2 | CDKN2A-deletion | Botelho - 2008^r80^ | pleural, peritoneal | 91 (30/3) | 55 (17/14) | 91 (30/3) | 100 (39/0) |
| 1.3 | CDKN2A-deletion | Illei - 2003^r78^ | pleural, peritoneal, pericardial | 92 (12/1) | 100 ^#^ (19/0) |  |  |

^#^(r78) values of other malignant diseases included also samples that were cytological negative for malignant cells;
